# TMEM106B regulates microglial proliferation and survival in response to demyelination

**DOI:** 10.1126/sciadv.add2676

**Published:** 2023-05-05

**Authors:** Tingting Zhang, Weilun Pang, Tuancheng Feng, Jennifer Guo, Kenton Wu, Mariela Nunez Santos, Akshayakeerthi Arthanarisami, Alissa L. Nana, Quynh Nguyen, Peter J. Kim, Joanna L. Jankowsky, William W. Seeley, Fenghua Hu

**Affiliations:** ^1^ Department of Molecular Biology and Genetics, Weill Institute for Cell and Molecular Biology, Cornell University, Ithaca, NY 14853, USA.; ^2^ Department of Neurology, University of California, San Francisco, CA 94158, USA.; ^3^ Department of Neuroscience, Huffington Center on Aging, Baylor College of Medicine, Houston, TX, USA.; ^4^ Departments of Molecular and Cellular Biology, Neurology, and Neurosurgery, Huffington Center on Aging, Baylor College of Medicine, Houston, TX, USA.; ^5^ Department of Pathology, University of California, San Francisco, CA 94158, USA.

## Abstract

TMEM106B, a lysosomal transmembrane protein, has been closely associated with brain health. Recently, an intriguing link between TMEM106B and brain inflammation has been discovered, but how TMEM106B regulates inflammation is unknown. Here, we report that TMEM106B deficiency in mice leads to reduced microglia proliferation and activation and increased microglial apoptosis in response to demyelination. We also found an increase in lysosomal pH and a decrease in lysosomal enzyme activities in TMEM106B-deficient microglia. Furthermore, TMEM106B loss results in a significant decrease in the protein levels of TREM2, an innate immune receptor essential for microglia survival and activation. Specific ablation of TMEM106B in microglia results in similar microglial phenotypes and myelination defects in mice, supporting the idea that microglial TMEM106B is critical for proper microglial activities and myelination. Moreover, the *TMEM106B* risk allele is associated with myelin loss and decreased microglial numbers in humans. Collectively, our study unveils a previously unknown role of TMEM106B in promoting microglial functionality during demyelination.

## Teaser

The dementia risk factor TMEM106B is essential for microglial survival and proliferation in response to demyelination.

## INTRODUCTION

Encoding transmembrane protein 106B (*TMEM106B*), was originally found as a risk factor for frontotemporal lobar degeneration (FTLD) with *GRN* mutations (*1*–*5*). *TMEM106B* has been recently involved in many other neurodegenerative diseases, including FTLD-*C9ORF72* (*5*–*8*), amyotrophic lateral sclerosis (*9*), Parkinson’s disease (*10*), Alzheimer’s disease (*11*), and limbic-predominant age-related TAR DNA binding protein-43 (TDP-43) encephalopathy (*12*). A heterozygous D252N mutation in *TMEM106B* causes hypomyelinating leukodystrophy (HLD) (*13*, *14*), a group of heritable neurodevelopmental disorders characterized by abnormal myelination in the central nervous system (CNS) (*15*). In addition, *TMEM106B* was found to be one of the main determinants of brain aging (*16*). More intriguingly, the C-terminal fragment of TMEM106B was recently found to form amyloid fibrils in the aged brain and several neurodegenerative diseases (*17*–*21*).

At molecular and cellular levels, TMEM106B is a type II transmembrane protein located within the late endosome/lysosome (*22*–*24*) and has been shown to affect lysosomal morphology and function (*22*–*25*), lysosome pH (*23*, *26*, *27*), lysosome exocytosis (*27*), lysosomal positioning with the cell (*28*), lysosome trafficking in neuronal dendrites (*29*), and lysosomal trafficking across the axon initial segment (AIS) in motor neurons (*30*, *31*). Thus, TMEM106B is critical for proper lysosomal function.

In the central nervous system (CNS), TMEM106B is ubiquitously expressed by many different cell types, with particularly high expression in neurons and oligodendrocytes (*32*). TMEM106B-deficient mice show lysosomal trafficking defects in the axons of motor neurons and Purkinje cells (*30*, *31*) and reduced survival of Purkinje cells during aging (*33*–*35*). TMEM106B deficiency also results in the perinuclear localization of lysosomes in oligodendrocytes, leading to trafficking defects of the main myelin membrane protein proteolipid protein (PLP) and myelination deficits (*28*, *36*).

Recent transcriptomic studies have revealed an intriguing link between *TMEM106B* and inflammation (*16*, *37*). *TMEM106B* was shown to modulate innate immune cell inflammatory polarization, CNS inflammation pathways, and degenerative changes independent of disease (*16*). A *TMEM106B* variant was shown to confer protection in the inflammatory late-onset Alzheimer’s disease in another study (*37*). CNS inflammation is mainly mediated by microglia, the resident immune cell, which constantly monitors the environment in the CNS. Dysregulated microglia activation plays a critical role in the pathogenesis of neurodegenerative diseases (*38*–*42*). However, how TMEM106B regulates microglial function and inflammation is unknown.

In this study, we show that TMEM106B deficiency leads to reduced microglial survival, proliferation, and activation in response to cuprizone (CPZ)– and lipopolysaccharide (LPS)–induced demyelination. Loss of TMEM106B results in severe lysosomal defects in microglia and decreased levels of triggering receptor expressed on myeloid cells 2 (TREM2), a transmembrane protein essential for microglia proliferation and survival (*41*, *43*, *44*). Microglial dysregulation and myelination defects were also observed in mice with TMEM106B specifically ablated in microglia. Furthermore, TMEM106B risk alleles are associated with increased myelin loss and decreased microglia density in the white matter in humans. Together, our results show that TMEM106B modulates CNS inflammation and myelination by regulating microglial survival in response to demyelination.

## RESULTS

### *Tmem106b^−/−^* mice have increased susceptibility to CPZ-induced demyelination and fail to remyelinate

**Fig. 1.**
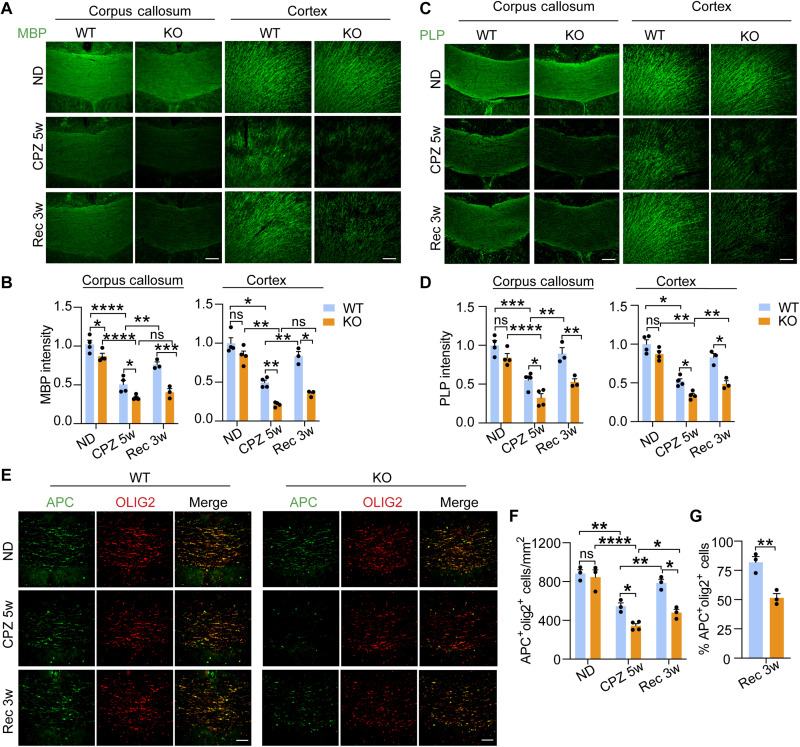
TMEM106B deficiency leads to increased demyelination after CPZ treatment and impaired remyelination during recovery. (**A** to **D**) Ten-week-old WT mice and *Tmem106b*^−/−^ mice (KO) mice were fed with normal chow (ND) or CPZ-containing chow for 5 weeks (CPZ 5w). Another group of mice was fed with normal chow for 3 weeks after 5 weeks of CPZ treatment (Rec 3w). Brain sections were stained with MBP and PLP antibodies. Representative images from the corpus callosum region and frontal cortex were shown. Scale bars, 100 μm. MBP and PLP intensities were quantified in (B) and (D), respectively. Data represent the means ± SEM. Statistical significance was analyzed by two-way ANOVA (*n* = 3 to 4 mice per group). ns, not significant; *P < 0.05; **P < 0.01; ***P < 0.001; ****P < 0.0001. (**E** to **G**) WT and KO mice were fed with normal chow for 3 weeks after 5 weeks of CPZ treatment (Rec 3w). Brain sections were stained with antibodies against APC and OLIG2. Representative images from the corpus callosum region were shown. Scale bars, 100 μm. The total number of mature oligodendrocytes (OLs) (APC^+^/OLIG2^+^) at different time points was quantified in (F). Data represent the means ± SEM. Statistical significance was analyzed by two-way ANOVA (*n* = 3 to 4 mice per group). *P < 0.05; **P < 0.01; ****P < 0.0001. The ratio of mature OLs (APC^+^/OLIG2^+^) and total OLs (OLIG2^+^) was quantified in (G). Data were analyzed by unpaired two-tailed Student’s *t *test (*n* = 3 mice per group). **P < 0.01.

Since TMEM106B is genetically linked to HLD, it is important to dissect its role in myelination. To do so, we treated wild-type (WT) and *Tmem106b^−/−^* mice with CPZ for 5 weeks to induce the apoptosis of mature oligodendrocytes and demyelination (*45*–*47*). Mice were then fed with a normal diet for 3 weeks to allow recovery and remyelination. After 5 weeks of treatment, CPZ-induced demyelination was detected in multiple brain regions of WT and *Tmem106b^−/−^* mice based on immunostaining of two major myelin proteins, myelin basic protein (MBP) and PLP, including the cortex, striatum, and most notably the corpus callosum region, as previously reported (*46*, *48*) (Fig. 1, A to D). However, a significantly increased loss of MBP and PLP signals was observed in *Tmem106b^−/−^* mice compared to WT mice after 5 weeks of CPZ treatment, supporting the idea that loss of TMEM106B results in increased susceptibility to CPZ-induced demyelination (Fig. 1, A to D). After 3 weeks of CPZ removal and recovery, MBP and PLP intensities were restored close to normal levels in the WT mice (Fig. 1, A to D), indicating successful remyelination. However, the reduction of MBP and PLP levels persists in TMEM106B-deficient mice 3 weeks after the removal of CPZ (Fig. 1, A to D). These results are consistent with a recently published study (*36*) and support the notion that TMEM106B deficiency leads to exacerbated demyelination in response to CPZ and a failure to regenerate myelin after CPZ removal.

To determine changes in oligodendrocytes during the course of demyelination and remyelination, we examined the densities of total and mature oligodendrocytes at different time points using Olig2 and adenomatous polyposis coli (APC) as a marker, respectively. Under normal conditions, no difference in the number of mature oligodendrocytes (APC^+^Olig2^+^) was found between WT and *Tmem106b^−/−^* mice (Fig. 1, E and F). After 5 weeks of CPZ treatment, a decrease in the number of mature oligodendrocytes was observed in both WT and *Tmem106b^−/−^* mice, and this reduction was significantly greater in *Tmem106b^−/−^* mice compared to WT mice (Fig. 1, E and F), suggesting increased death of mature oligodendrocytes in *Tmem106b^−/−^* mice upon CPZ treatment. In addition, a decreased ratio of mature oligodendrocytes (APC^+^Olig2^+^) to total oligodendrocytes (Olig2^+^) was observed in *Tmem106b^−/−^* mice compared with WT mice after 3 weeks of recovery (Fig. 1G), indicating impaired differentiation of oligodendrocyte precursor cells (OPCs) in *Tmem106b^−/−^* mice during the recovery phase.

### TMEM106B deficiency leads to decreased microglia proliferation, survival, and activation in response to demyelination

**Fig. 2.**
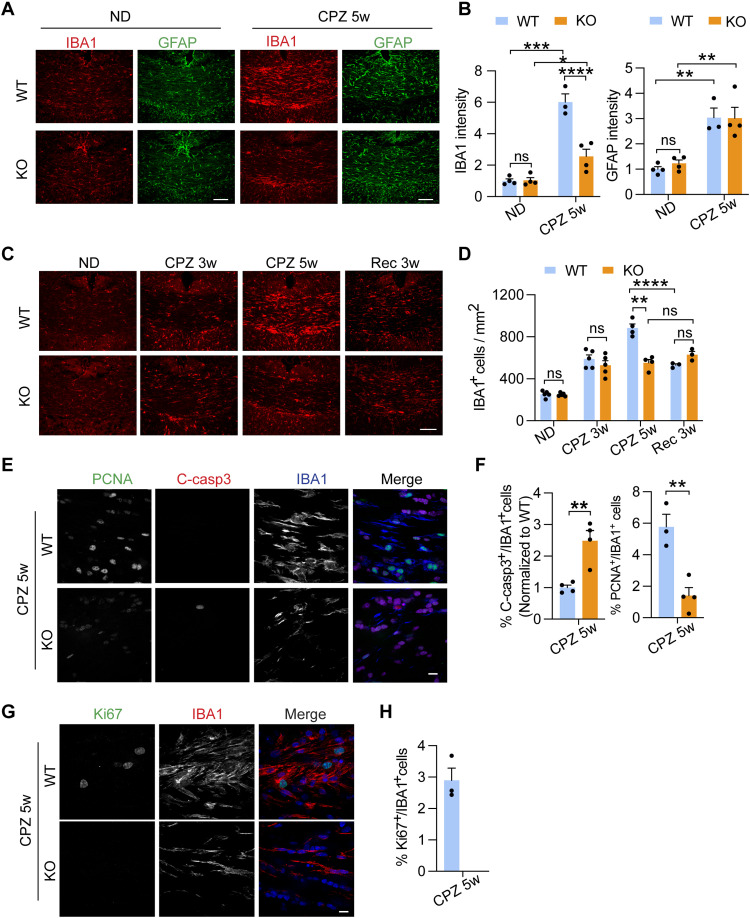
TMEM106B deficiency leads to decreased microglia proliferation and survival in response to demyelination. (**A** and **B**) WT and KO mice were fed with normal chow (ND) or CPZ-containing chow for 5 weeks (CPZ 5w). Brain sections were stained with IBA1 and GFAP antibodies. Representative images from the corpus callosum (CC) region were shown (A). Scale bars, 100 μm. IBA1 and GFAP levels were quantified in (B). Data represent the means ± SEM. Statistical significance was analyzed by two-way ANOVA (*n* = 3 to 4 mice per group). (**C** and **D**) Brain sections from WT and KO mice receiving ND, CPZ 5w, and Rec 3w treatments were stained with IBA antibodies. Representative images from the CC were shown (C). Scale bar, 100 μm. The numbers of IBA1^+^ microglia/mm^2^ in the CC were quantified (D). Data represent the means ± SEM. Statistical significance was analyzed by two-way ANOVA (*n* = 3 to 5 mice per group). (**E** and **F**) Brain sections from WT and KO mice treated with CPZ for 5 weeks were stained with PCNA, cleaved caspase3 (C-casp3), and IBA1 antibodies. Representative images from CC were shown (E). Scale bar, 10 μm. The percentage of c-Casp3^+^ microglia or PCNA^+^ microglia were quantified (F). Data represent the means ± SEM. Statistical significance was analyzed by unpaired two-tailed Student’s *t* test (*n* = 4 mice per group). (**G** and **H**) Brain sections from WT and KO mice treated with CPZ for 5 weeks were stained with Ki67 and IBA1 antibodies. Representative images from CC were shown (G). Scale bar, 10 μm. The percentage of Ki67^+^ microglia was quantified (H). Statistical significance was analyzed by unpaired two-tailed Student’s *t* test (*n* = 3 to 5 mice per group). Data represent the means ± SEM. *P < 0.05; **P < 0.01; ***P < 0.001; ****P < 0.0001.

CPZ-induced death of oligodendrocytes is known to result in the accumulation of myelin debris and activation of astrocytes and microglia (*46*, *47*, *49*). While astrocyte proliferation in the corpus callosum region, as shown by glial fibrillary acidic protein (GFAP) intensity, is comparable in WT and *Tmem106b^−/−^* mice, a significant decrease in the total intensity of microglial marker ionized calcium-bingding adaptor molecule 1 (IBA1) was observed in *Tmem106b^−/−^* mice after 5 weeks of CPZ treatment (Fig. 2, A and B), indicating a specific defect in microglia, but not astrocyte, activation in response to demyelination. Microglia play an important role in myelination: They not only help remove damaged myelin debris but also promote the differentiation of OPCs and subsequent remyelination (*50*–*52*). In WT mice, an increase in the number of IBA1-positive microglia starts at 3 weeks after CPZ treatment and continues at 5 weeks (Fig. 2, C and D). A similar increase in microglial numbers was found in *Tmem106b^−/−^* mice 3 weeks after CPZ treatment (Fig. 2, C and D). However, after 5 weeks of CPZ treatment, the number of microglia is significantly lower in *Tmem106b^−/−^* mice compared to WT mice (Fig. 2, C and D). In addition, after 3 weeks of recovery, the number of IBA1^+^ microglia in the corpus callosum region of WT mice is greatly reduced (Fig. 2, C and D). However, this decrease is not observed in *Tmem106b^−/−^* mice (Fig. 2, C and D). These data suggest that the kinetics of microglial activation in response to demyelination is altered by TMEM106B loss.

Decreased number of microglia in *Tmem106b^−/−^* mice after 5 weeks of CPZ treatment could result from a decrease in microglia proliferation, an increase in apoptosis, or both. Previous studies have reported that the CPZ-induced increase in the number of microglia in the corpus callosum region is mainly due to local microglial proliferation (*53*, *54*). To determine whether TMEM106B loss affects microglial proliferation, we determined the number of proliferating microglia using coimmunostaining of IBA1 and proliferating cell nuclear antigen (PCNA) or Ki67, markers for cell proliferation. We found that microglia proliferation is significantly decreased in *Tmem106b^−/−^* mice, as shown by the percentage of PCNA^+^ microglia (Fig. 2, E and F). This is also confirmed by Ki67 and IBA1 costaining. In WT mice, Ki67-positive microglia account for 2.9 ± 0.4% of all IBA1-positive microglia after 5-week CPZ treatment. However, we fail to detect any Ki67- and IBA1-positive cells in *Tmem106b^−/−^* mice (Fig. 2, G and H). To determine whether TMEM106B loss also induces microglial apoptosis, we costained cleaved caspase-3 with IBA1 and detected an elevated percentage of IBA1^+^ microglia with positive cleaved caspase-3 signals in the corpus callosum region of *Tmem106b^−/−^* mice after 5 weeks of CPZ treatment (Fig. 2, E and F). Together, these results show that TMEM106B deficiency results in reduced microglial proliferation and increased microglial apoptosis in response to demyelination.

**Fig. 3.**
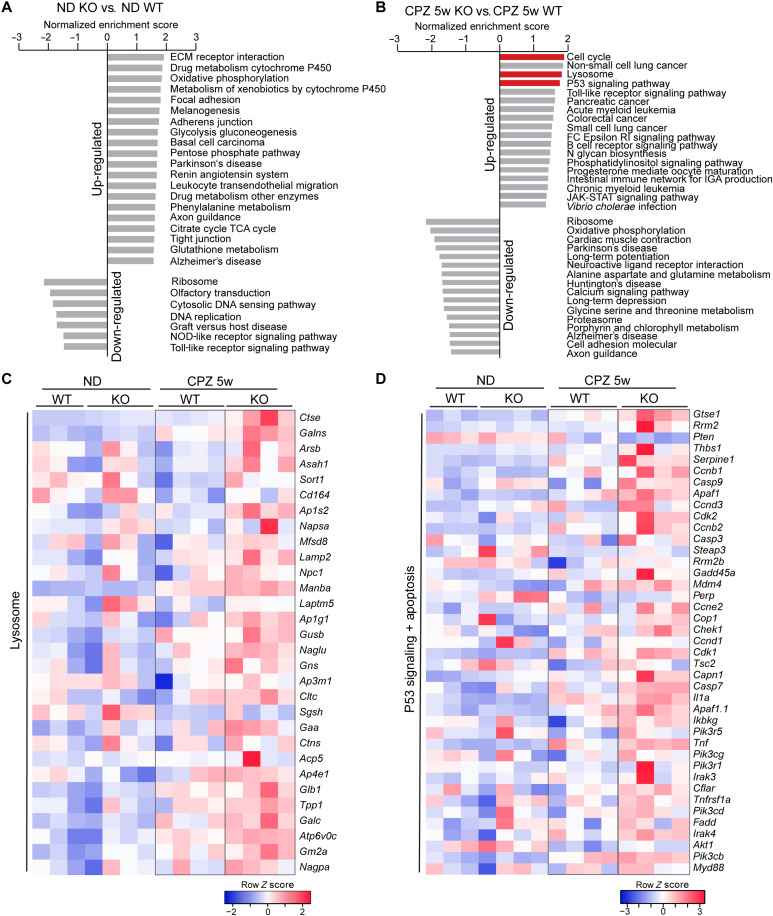
TMEM106B deficiency leads to transcriptomic alterations in microglia. (**A** and **B**) WT and KO mice (*n* = 3 to 4 for each condition) were fed with normal chow (ND) or CPZ-containing chow for 5 weeks (CPZ 5w). Microglia were isolated from the brain, and total RNAs were extracted for RNA-seq analysis. Normalized enrichment scores (NESs) were calculated for the GSEA using the Kyoto Encyclopedia of Genes and Genomes (KEGG) gene sets. Top up-regulated and down-regulated gene sets with FDR < 0.25 or nominal *P* value <0.05 are listed. The lysosome, cell cycle, and p53 signaling pathways are highlighted in red. (**C** and **D**) Heatmap illustrating core enrichment gene expression changes in the lysosome (C) and p53 and apoptosis (D) pathways based on GSEA of CPZ-treated WT and KO microglia samples.

To further dissect the function of TMEM106B in microglia, we compared gene expression profiles of CD11b^+^ microglia isolated from WT and *Tmem106b^−/−^* mouse brains with or without CPZ treatment (Fig. 3, A and B, and fig. S1). While only 29 differentially expressed genes (DEGs) with normalized counts >50 and false discovery rate (FDR) <0.05 were detected in TMEM106B-deficient microglia compared to WT microglia without CPZ treatment, a total of 870 DEGs were identified between microglia isolated from CPZ-treated WT and *Tmem106b^−/−^* mice (datasets S1 and S2 and fig. S2). Cell cycle, lysosome, and p53 signaling pathways were identified as the top significantly enriched pathways after gene set enrichment analysis (GSEA) (Fig. 3, B to D, and dataset S3), consistent with our immunostaining analysis results showing that TMEM106B loss results in decreased microglial proliferation and increased apoptosis.

### TMEM106B deficiency leads to lysosomal defects and microglial activation deficits in response to demyelination

**Fig. 4.**
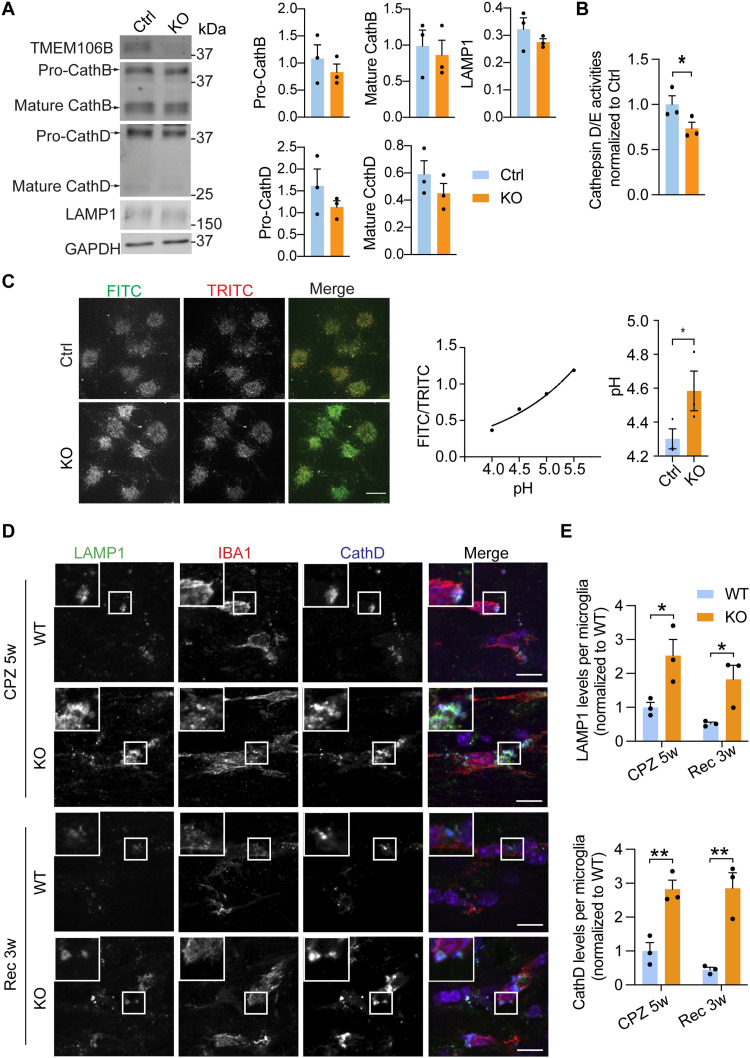
TMEM106B deficiency results in lysosomal abnormalities in microglia. (**A**) Western blot analysis of lysosomal proteins in the control and *Tmem106b^−/−^* BV2 cells using the indicated antibodies. The levels of each protein were normalized to glyceraldehyde-3-phosphate dehydrogenase (GAPDH). Data from three independent experiments were plotted. (**B**) Reduced CathD/E activities in TMEM106B-deficient BV2 cells. CathD/E activities were measured in control and *Tmem106b^−/−^* BV2 cells using fluorogenic substrates. Data represent the means ± SEM. Statistical significance was analyzed by unpaired one-tailed Student’s *t* test (*n* = 3). *P < 0.05. (**C**) Lysosomal pH measurement in control and *Tmem106b^−/−^* BV2 cells. BV2 cells were incubated with dextran conjugated with pH-sensitive fluorescein (green) and pH-insensitive tetramethylrhodamine (red). Representative images were shown. Scale bar, 10 μm. FITC and TRITC intensity per cell were quantified and the ratio (FITC/TRITC) was calculated. Lysosomal pH was determined from the calibration curve generated from the FTIC/TRITC value of permeabilized cells in various calibration standard solutions of different pH values. Data represent the means ± SEM. Statistical significance was analyzed by unpaired one-tailed Student’s *t* test (*n* = 3). *P < 0.05. (**D** and **E**) WT and KO mice were fed with CPZ-containing chow (CPZ 5w) for 5 weeks or fed with CPZ for 5 weeks and a normal diet for additional 3 weeks after CPZ removal (Rec 3w). Brain sections were stained with LAMP1, CathD, and IBA1 antibodies. Representative confocal images in the corpus callosum region were shown (A). Scale bars, 10 μm. The LAMP1 or CathD levels in microglia were quantified (B). Data represent the means ± SEM. Statistical significance was analyzed by unpaired two-tailed Student’s *t* test (*n* = 3 mice per group). *P < 0.05 and **P < 0.01.

TMEM106B is known to be critical for proper lysosomal function (*55*). In line with this, we found that the lysosome pathway is significantly altered in CD11b^+^ adult microglia upon TMEM106B loss in response to CPZ treatment in our RNA sequencing (RNA-seq) analysis (Fig. 3, B and C). To determine the effect of TMEM106B on lysosome in microglia, we ablated TMEM106B in the microglial cell line BV2 (Fig. 4A). TMEM106B deletion results in a slight but not significant reduction in the levels of lysosomal proteins cathepsin B (CathB), cathepsin D (CathD), and lysosomal-associated membrane protein 1 (LAMP1) (Fig. 4A). However, the activities of lysosomal proteases CathD/E are significantly reduced in *Tmem106b^−/−^* BV2 cells (Fig. 4B). Since lysosomal pH is critical for lysosomal enzymatic activities, and TMEM106B is known to interact with V–adenosine triphosphatase (ATPase) to regulate lysosomal pH (*23*, *26*, *27*), we measured lysosomal pH in control and *Tmem106b^−/−^* BV2 cells and found increased lysosomal pH in *Tmem106b^−/−^* cells (Fig. 4C).

To further investigate the effect of TMEM106B on microglial lysosomes in vivo, we immunostained brain sections with antibodies against LAMP1, a lysosome membrane protein, and CathD, a lysosome proteinase. We found that lysosomes are frequently enlarged, and the levels of LAMP1 and CathD are significantly increased in *Tmem106b^−/−^* microglia in response to CPZ-induced demyelination (Fig. 4, D and E). In addition, this defect persists 3 weeks after CPZ removal, indicating thatlysosomes are severely altered in *Tmem106b^−/−^* microglia and cannot return to their normal state in response to myelin debris.

**Fig. 5.**
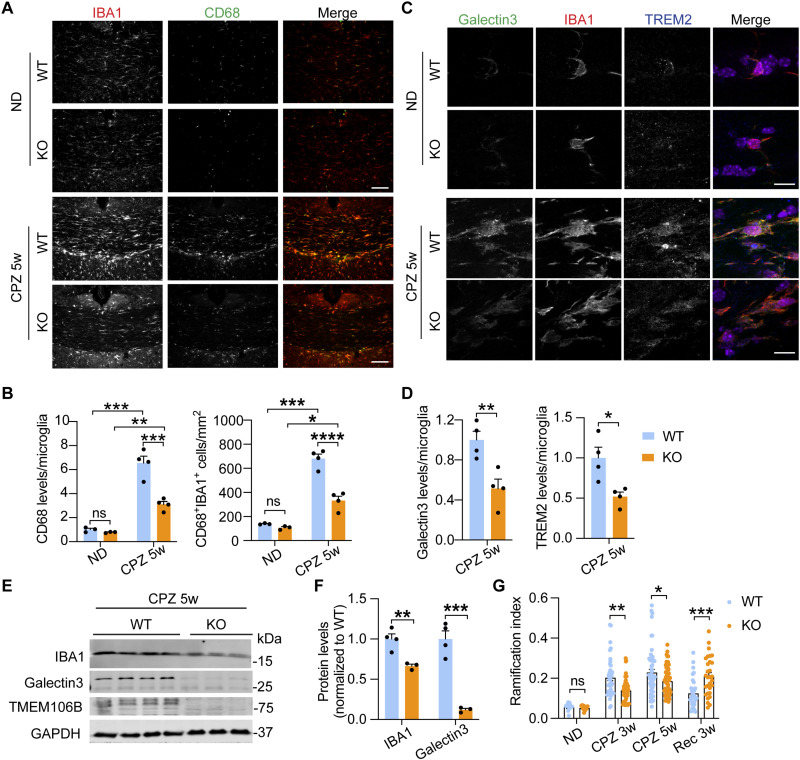
TMEM106B deficiency leads to reduced microglial activation in response to demyelination. (**A** and **B**) Brain sections from WT and KO mice untreated or treated with CPZ for 5 weeks were stained with IBA1 and CD68 antibodies. Representative images in the CC region were shown (A). Scale bars, 100 μm. CD68 levels per microglia and the number of CD68^+^IBA1^+^ microglia were quantified (B). Data represent the means ± SEM. Statistical significance was analyzed by two-way ANOVA (*n* = 3 to 4 mice per group). (**C** and **D**) Brain sections from WT and KO mice untreated or treated with CPZ for 5 weeks were stained with Galectin-3, TREM2, and IBA1 antibodies. Representative images in CC were shown (C). Scale bars, 10 μm. Galectin-3 and TREM2 levels per microglia were quantified (D). Data represent the means ± SEM. Statistical significance was analyzed by unpaired two-tailed Student’s *t* test (*n* = 4 mice per group). (**E** and **F**) Western blot analysis of IBA1 and Galectin3 in the cortical lysates from WT and KO mice fed with CPZ for 5 weeks. The protein levels were quantified and normalized to GAPDH. Data represent the means ± SEM. Statistical significance was analyzed by unpaired two-tailed Student’s *t* test (*n* = 3 to 4 mice per group). (**G**) Microglia morphology analysis at the different time points was done using a ramification index [RI = 4π × cell area/(cell perimeter)^2^] that describes microglial cell shape. Ten microglia were analyzed for mice fed with a normal diet. A total of 40 to 70 cells per group from three to five independent mice per group at different time points were analyzed. Data represent the means ± SEM. Statistical significance was analyzed by unpaired two-tailed Student’s *t* test. *P < 0.05; **P < 0.01; ***P < 0.001; ****P < 0.0001.

Next, we determined whether microglial activation status in response to demyelination is affected by TMEM106B deficiency. The expression of several disease-associated microglia (DAM) markers, including *Trem2*, *Lgals3*, and *Gpnmb*, which are known to be up-regulated in response to demyelination (*56*, *57*), is lower in *Tmem106b^−/−^* microglia compared to WT control upon CPZ treatment as revealed by RNA-seq analysis (fig. S5, A and B). On the other hand, the expression of homeostatic genes, *P2ry12* and *P2ry13*, is higher in *Tmem106b^−/−^* microglia compared to WT control (fig. S5A). This is further confirmed by immunostaining. The cellular intensity of CD68, a protein associated with microglial activation, and the number of CD68^+^ microglia are significantly increased in microglia in response to CPZ treatment in both WT and *Tmem106b^−/−^* mice. However, this response is significantly diminished in CPZ-treated *Tmem106b^−/−^* mice compared to WT mice (Fig. 5, A and B). In addition, cellular protein levels of DAM markers, TREM2 and Galectin-3 (encoded by the *Lgals3* gene), are also much reduced in *Tmem106b^−/−^* mice, as compared to WT mice under CPZ-treated conditions (Fig. 5, C to F) (fig. S3B). Alterations in microglial activation are also reflected by changes in microglia morphology as shown by ramification index (RI) quantification (*58*, *59*). Under basal conditions, *Tmem106b^−/−^* microglia do not show any alteration in morphology (Fig. 5G). However, upon CPZ treatment, *Tmem106b^−/−^* microglia are much more ramified compared to WT control, suggesting that they are less activated. In addition, after CPZ removal, WT microglia return to the basal state with increased ramification, but *Tmem106b^−/−^* microglia remain in amoeba-like activated states (Fig. 5G). Together, these data support the idea that TMEM106B deficiency leads to impaired microglial activation in response to demyelination and alterations in microglial dynamics during the recovery phase.

### Decreased microglial proliferation and microgliosis in *Tmem106b^−/−^* mice in response to LPS

**Fig. 6.**
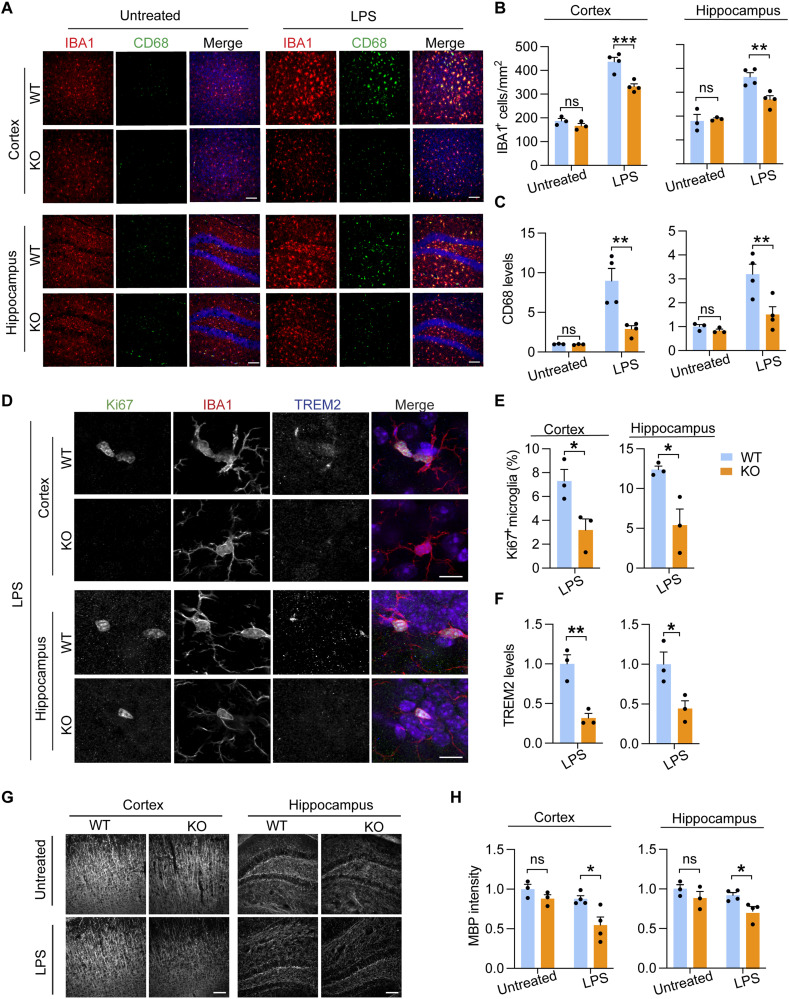
TMEM106B deficiency results in decreased microglial proliferation and activation in response to LPS. (**A** to **C**) Four-month-old WT and KO mice were untreated or treated with LPS intracerebroventricularly. Brains were harvested 72 hours after injection, and brain sections were stained with IBA1 and CD68 antibodies. Representative images from the cortex and dentate gyrus of the hippocampus were shown (A). Scale bar, 100 μm. The number of IBA1^+^ microglia (B) and CD68 levels per microglia (C) were quantified in the cortex and hippocampus for the experiment. Data represent the means ± SEM. Statistical significance was analyzed using two-way ANOVA (*n* = 3 to 4 mice per group). **P < 0.01 and ***P < 0.001. (**D** to **F**) Brain sections from LPS-treated WT and KO mice were stained with Ki67, TREM2, and IBA1 antibodies. Representative images from the cortex and dentate gyrus of the hippocampus were shown (D). Scale bar, 10 μm. The number of Ki67^+^ microglia (E) and TREM2 levels per microglia (F) in the cortex and hippocampus region were quantified. Data represent the means ± SEM. Statistical significance was calculated using the unpaired two-tailed Student’s *t t*est (*n* = 3 mice per group). *P < 0.05; **P < 0.01. (**G** and **H**) Brain sections from untreated or LPS-treated WT and KO mice were stained with MBP antibodies. Representative images from the cortex and dentate gyrus of the hippocampus were shown (G). Scale bars, 100 μm. MBP intensities were quantified in (H). Data represent the means ± SEM. Statistical significance was analyzed by two-way ANOVA (*n* = 3 to 4 mice per group). *P < 0.05.

To investigate the role of TMEM106B in regulating microglial responses to other inflammatory stimuli, we treated WT and *Tmem106b^−/−^* mice with lipopolysaccharide (LPS), a Toll-like receptor 4 agonist, to induce acute CNS inflammation (*60*) and demyelination (*61*, *62*). Mice were injected with LPS intracerebroventricularly. We found that LPS treatment resulted in a notable increase in the number of activated microglia in the cortex and hippocampus of WT mouse brain; however, this response was significantly reduced in *Tmem106b^−/−^* mice, based on IBA1 and CD68 staining (Fig. 6, A to C). To determine whether the reduction in microglia number in *Tmem106b^−/−^* mice is due to a decrease in microglia proliferation, we co-immunostained brain sections with antibodies against IBA1 and Ki67 and quantified the number of cells positive for both Ki67 and IBA1. *Tmem106b^−/−^* mice show a marked decrease in microglia proliferation in the cortex and hippocampus after LPS treatment (Fig. 6, D to F). Furthermore, decreased levels of CD68 and TREM2 in microglia were found in *Tmem106b^−/−^* mice compared to WT mice after LPS treatment both in the cortex and in the hippocampus (Fig. 6, A, C, D, and F), supporting the idea that TMEM106B deficiency leads to impaired microglial activation in response to LPS. In addition, consistent with a previous report (*62*), we found that LPS injection results in mild demyelination in WT mice, as shown by decreased MBP intensity. This phenotype is exacerbated in TMEM106B-deficient mice, leading to a ~50% reduction in MBP signals with LPS treatment (Fig. 6, G and H).

### Microglia-specific ablation of TMEM106B results in decreased microglia viability and myelination defects

**Fig. 7.**
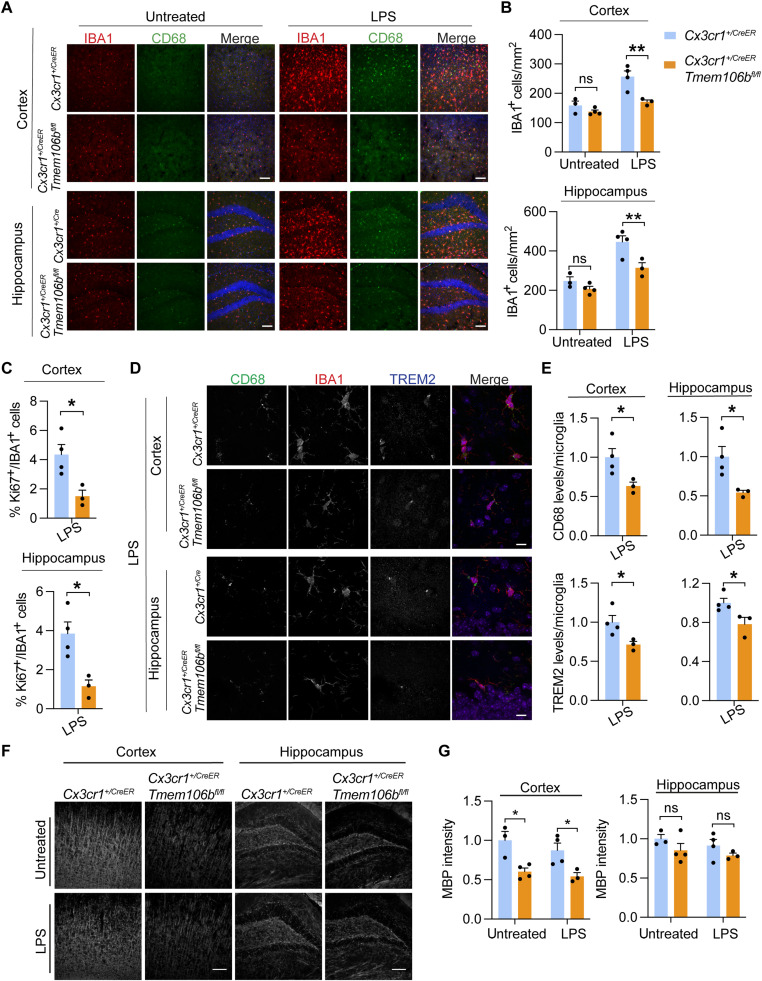
Microglia-specific ablation of TMEM106B results in decreased microglial proliferation and activation in response to LPS. (**A** to **C**) *Cx3cr1^+/CreER^ Tmem106b ^flox/flox^* mice and *Cx3cr1^+/CreER^* mice were fed with tamoxifen for 2 weeks at 4 weeks of age and untreated or treated with LPS intracerebroventricularly at 12 weeks of age. Brains were harvested 72 hours after injection, and brain sections were stained with IBA1 and CD68 antibodies. Representative images from the cortex and dentate gyrus of the hippocampus were shown (A). Scale bars, 100 μm. The number of IBA1^+^ microglia was quantified (B). Data represent the means ± SEM. Statistical significance was analyzed using two-way ANOVA (*n* = 3 to 4 mice per group). (C) Brain sections from experiments described in (A) were stained with Ki67 and IBA1 antibodies, and the percentage of Ki67^+^IBA1^+^ microglia in the cortex and hippocampus region after LPS treatment was quantified. Data represent the means ± SEM; unpaired two-tailed Student’s *t* test (*n* = 3 to 4 mice per group). (**D** and **E**) Brain sections from LPS-treated control and *106b^fl/fl^* mice were stained with CD68, TREM2, and IBA1 antibodies. Representative images from the cortex and dentate gyrus of the hippocampus were shown (D). Scale bars, 10 μm. The levels of CD68 and TREM2 per microglia were quantified (E). Data represent the means ± SEM; unpaired two-tailed Student’s *t* test (*n* = 3 mice per group). (**F** and **G**) Brain sections from untreated or LPS-treated control and *106b^fl/fl^* mice were stained with MBP antibodies. Representative images from the cortex and dentate gyrus of the hippocampus were shown (F). Scale bars, 100 μm. MBP intensities were quantified in (G). Data represent the means ± SEM; two-way ANOVA (*n* = 3 to 4 mice per group). *P < 0.05 and **P < 0.01.

The alterations in microglial dynamics in TMEM106B-deficient conditions observed in vivo could be due to changes in myelination since TMEM106B has been shown to function in oligodendrocytes to regulate lysosomal positioning and PLP trafficking (*28*, *36*). To further elucidate the function of TMEM106B in microglia, we specifically deleted TMEM106B in microglia by expressing the tamoxifen-inducible Cre recombinase under the control of the CX3CR1 promoter (*Cx3cr1^+/CreER^*) (*63*). *Cx3cr1^+/CreER^ Tmem106b ^flox/flox^* mice were fed tamoxifen to activate Cre-mediated recombination specifically in microglia (fig. S4A). Microglial-specific ablation of TMEM106B is confirmed by Western blot analysis of CD11b^+^ microglia isolated from *Tmem106b ^flox/flox^* and *Cx3cr1^+/CreER^ Tmem106b ^flox/flox^* mice fed with tamoxifen. TMEM106B protein is specifically lost in CD11b^+^ microglia but no other brain cells in *Cx3cr1^+/CreER^ Tmem106b ^flox/flox^* mice (fig. S4B). To determine TMEM106B functions in microglia, *Cx3cr1^+/CreER^* and *Cx3cr1^+/CreER^ Tmem106b ^flox/flox^* mice were injected with LPS intracerebroventricularly. We found that specific ablation of TMEM106B in microglia results in similar defects in microglial proliferation and activation, as shown by Ki67, TREM2, IBA1, and CD68 staining (Fig. 7, A to E), indicating that loss of microglial TMEM106B is sufficient to cause microglial phenotypes seen in whole-body knockout. To determine whether deficiency of TMEM106B induces myelination defect, we immunostained brain sections with MBP antibodies. We found a significant reduction in MBP intensities in the *Cx3cr1^+/CreER^ Tmem106b ^flox/flox^* mice compared to *Cx3cr1^+/CreER^* mice under tamoxifen fed conditions, even without LPS treatment (Fig. 7, F and G). This result indicates that TMEM106B deficiency in microglia combined with haploinsufficiency of CX3CR1 leads to a severe defect in myelination.

### TMEM106B deficiency leads to decreased viability and increased cell death in cultured microglia

**Fig. 8.**
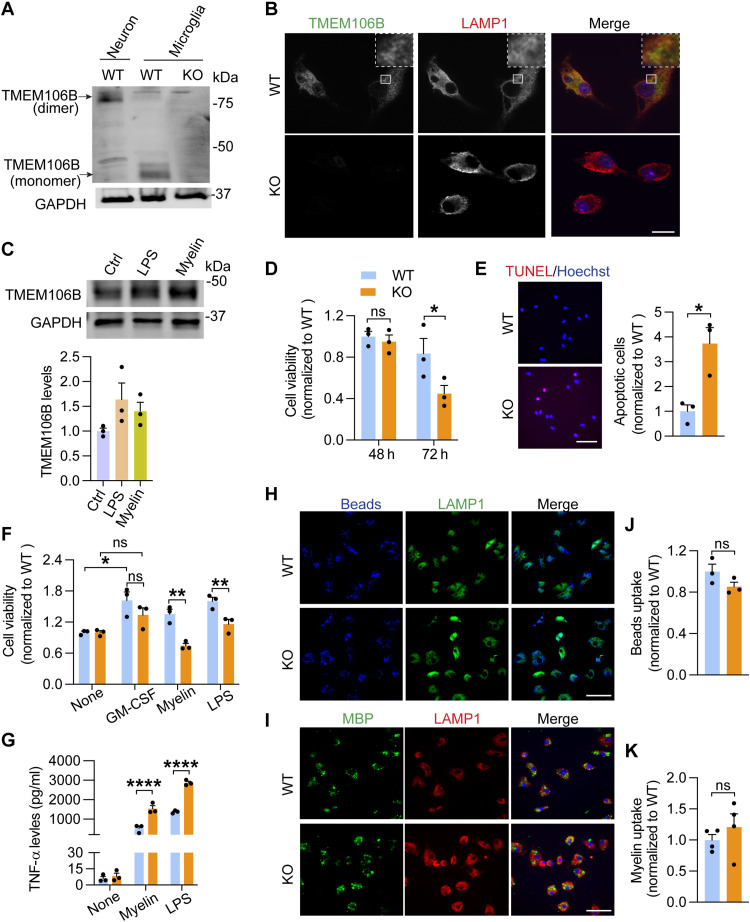
TMEM106B deficiency in microglia leads to decreased cell viability and enhanced inflammation. (**A**) Western blot analysis of primary cortical neuron and microglia lysates as indicated. Lysates were kept cold to allow the detection of TMEM106B dimers. (**B**) WT and KO microglia were stained with TMEM106B and LAMP1 antibodies. Scale bar, 10 μm. (**C**) WT primary microglia were untreated or treated with myelin or LPS treatment for 24 hours. TMEM106B levels relative to GAPDH were quantified from three independent samples. (**D**) WT and KO primary microglia were grown in cell culture for the indicated times, and the number of microglia was quantified. Data represent the means ± SEM; two-way ANOVA (*n* = 3). (**E**) WT and KO primary microglia 72 hours after plating were analyzed for apoptosis using TUNEL staining. Scale bar, 50 μm. The percentage of apoptotic cells was quantified. Data represent the means ± SEM; unpaired two-tailed Student’s *t* test (*n* = 3). (**F**) WT and KO primary microglia were treated with GM-CSF, myelin, or LPS for 24 hours. The number of microglia was quantified. Data represent the means ± SEM; two-way ANOVA (*n* = 4). (**G**) WT and KO primary microglia were either untreated or treated with myelin and LPS for 24 hours and TNF-α levels in the medium were quantified. Data represent the means ± SEM; two-way ANOVA (*n* = 3). (**H** to **K**) WT and KO primary microglia were fed with fluorescence-labeled Latex beads (H) or myelin (I). Cells were stained with LAMP1 and MBP antibodies as indicated. Scale bar, 50 μm. The fluorescence intensities from beads (H) or MBP (I) per cell were quantified. Data represent the means ± SEM; unpaired two-tailed Student’s *t* test (*n* = 3 to 4 independent experiments). *P < 0.05; **P < 0.01; ***P < 0.001; ****P < 0.0001.

To further study the function of TMEM106B in microglia, we cultured primary microglia from WT and *Tmem106b^−/−^* mice. We found that TMEM106B is expressed in the microglia (Fig. 8A) and localizes in the lysosomal compartments, as shown by colocalization with the lysosome marker LAMP1 (Fig. 8B). In addition, while TMEM106B is known to form SDS-resistant dimers in other cells, such as neurons (*22*–*25*) and CD11b^+^ microglia isolated from the adult brain (fig. S4B), this dimerization was not observed in cultured primary microglia (Fig. 8A), indicating that the dimerization status of TMEM106B might differ in cultured primary microglia from neonatal pups. Furthermore, the protein levels of TMEM106B increase upon LPS or myelin treatment (Fig. 8C). All these observations suggest that TMEM106B might regulate microglial behavior. To determine how TMEM106B affects microglia proliferation and survival, we first compared the survival of WT and *Tmem106b^−/−^* microglia under different treatment conditions. *Tmem106b^−/−^* microglia have significantly reduced viability 72 hours after plating compared to WT microglia (Fig. 8D). Increased numbers of terminal deoxynucleotidyl transferase–mediated deoxyuridine triphosphate nick end labeling (TUNEL)–positive apoptotic cells were found in *Tmem106b^−/−^* microglia compared with WT microglia (Fig. 8E), indicating that TMEM106B deficiency results in increased apoptosis of microglia. In addition, *Tmem106b^−/−^* microglia have reduced viability in response to myelin or LPS treatment (Fig. 8F). Together, our data support the notion that TMEM106B deficiency leads to enhanced microglial apoptosis in cell culture.

Next, we assessed whether microglial inflammatory responses in response to myelin or LPS are altered by TMEM106B loss by measuring secreted levels of the proinflammatory cytokine tumor necrosis factor–α (TNF-α). We found that TMEM106B deficiency leads to a significant increase in TNF-α secretion in response to both myelin and LPS treatment (Fig. 8G). However, no significant difference in the uptake rate of Latex beads or myelin debris was found between WT and *Tmem106b^−/−^* microglia (Fig. 8, H to K).

### TMEM106B regulates TREM2 levels

**Fig. 9.**
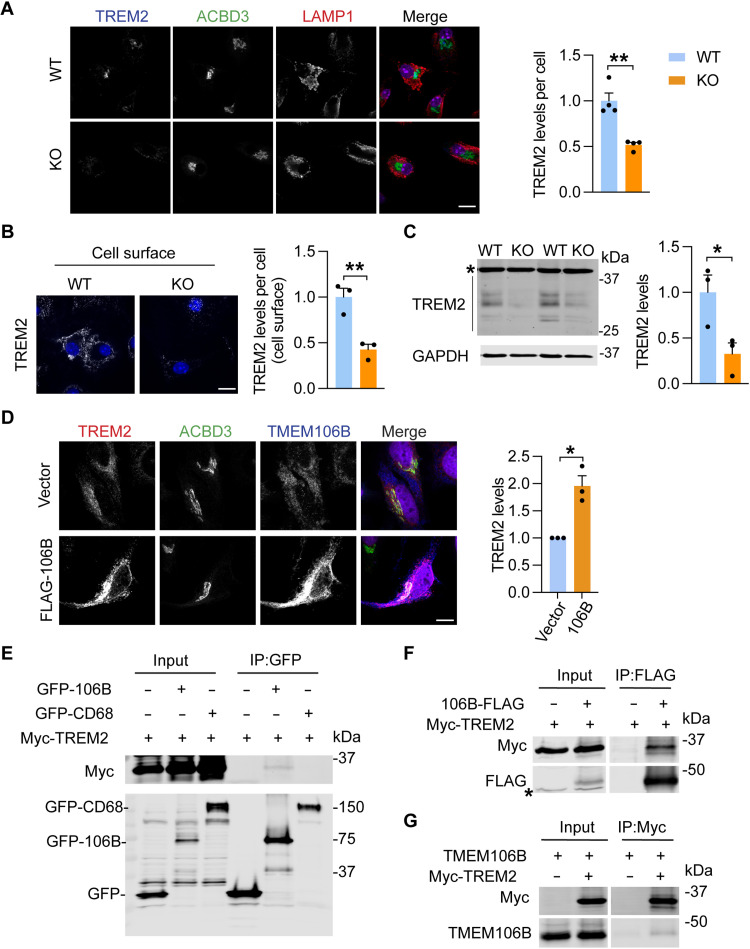
TMEM106B deficiency in microglia leads to decreased protein levels of TREM2. (**A** and **B**) Fixed (A) or live (B) WT and KO primary microglia were stained with the indicated antibodies. Scale bar, 10 μm. TREM2 levels per cell were quantified for a total of 142 WT microglia and 122 KO microglia from four independent batches of microglial culture (A) or a total of 86 WT microglia and 74 KO microglia from three independent batches of microglial culture (B). Data represent the means ± SEM. Statistical significance was analyzed by unpaired two-tailed Student’s *t* test [*n* = 4 (A) or *n* = 3 (B)]. **P < 0.01. (**C**) Western blot analysis of TREM2 levels in the microglia cultured from WT and KO mice. The protein levels were quantified and normalized to GAPDH. Data represent the means ± SEM. Statistical significance was analyzed by unpaired two-tailed Student’s *t* test (*n* = 3 independent batches of microglial culture). *P < 0.05. Asterisk indicated the nonspecific band. (**D**) HeLa cells stably expressing TREM2 were transfected with FLAG-tagged TMEM106B or control vector and stained with TREM2, ACBD3, and TMEM106B antibodies. Scale bar, 10 μm. TREM2 levels were quantified and normalized to vector control. Data represent the means ± SEM. Statistical significance was analyzed by one-sample *t* test (*n* = 3 independent experiments). *P < 0.05. (**E** to **G**) Myc-tagged TREM2 were cotransfected with GFP-tagged TMEM106B, GFP-tagged CD68, or GFP-expressing constructs (E) or cotransfected with FLAG-tagged TMEM106B or empty vector (F) into HEK293T cells as indicated. Untagged TMEM106B was cotransfected with Myc-TREM2 or empty vector into HEK293T cells as indicated (G). Cells were harvested 48 hours after transfection, and lysates were subjected to immunoprecipitations with anti-GFP (E), anti-FLAG (F), or anti-Myc (G) antibodies. IP products were analyzed by Western blots using the indicated antibodies.

Since TREM2 is a key regulator of microglia proliferation and survival (*41*, *43*, *44*), we next analyzed whether TMEM106B regulates TREM2 protein homeostasis to affect microglia. A significant decrease in TREM2 levels was observed in *Tmem106b^−/−^* microglia in CPZ- and LPS-treated mice compared to WT control (Figs. 5D and 6F). This was confirmed in cultured primary WT and *Tmem106b^−/−^* microglia, as demonstrated by a significant reduction in the levels of total and cell surface of TREM2 in *Tmem106b^−/−^* microglia (Fig. 9, A to C). While the mechanism by which TMEM106B regulates TREM2 levels remains to be fully determined, data from our RNA-seq analysis indicate a reduction in TREM2 mRNA levels in *Tmem106b^−/−^* microglia in both untreated and CPZ-treated conditions (fig. S5), indicating that TMEM106B could regulate TREM2 at the transcription level. In addition, we found that when TMEM106B is coexpressed with TREM2 in HeLa cells, TREM2 protein levels are dramatically increased compared to controls (Fig. 9D). This indicates that TMEM106B might stabilize TREM2, although we cannot rule out the possibility that this effect might be due to lysosomal dysfunction caused by TMEM106B overexpression. To determine whether TMEM106B physically interacts with TREM2, we performed coimmunoprecipitation experiments. We cotransfected human embryonic kidney (HEK) 293T cells with Myc-tagged human TREM2 (Myc-TREM2) and green fluorescent protein (GFP)–TMEM106B or GFP control plasmids. CD68, another lysosomal transmembrane protein highly expressed in microglia, was used as a control. We found that TREM2 weakly coimmunoprecipitates with GFP-tagged TMEM106B, but not CD68 (Fig. 9E). The interaction is further confirmed with immunoprecipitation (IP) using C-terminally FLAG-tagged TMEM106B (Fig. 9F) and with the reciprocal IP using anti-Myc antibodies to IP TREM2 (Fig. 9G). However, we failed to detect an interaction between TREM2 and TMEM106B at endogenous levels, suggesting that the binding between these two proteins could be weak or transient.

### TMEM106B polymorphisms affect microglia and myelination phenotypes in humans

**Fig. 10.**
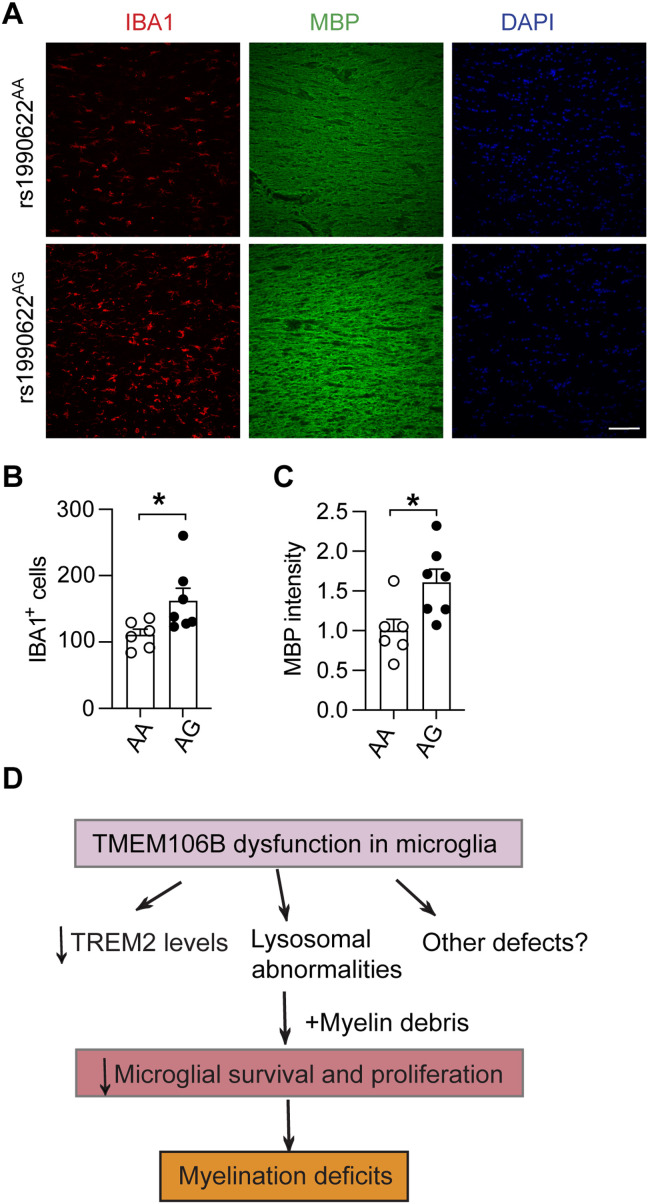
TMEM106B risk allele is associated with increased myelin loss and reduced microglial numbers in humans. (**A** to **C**) Corpus callosum sections of postmortem human brain tissues (table S1) were stained with MBP and IBA1 antibodies. Scale bar, 100 μm. Five to ten 20× images were taken from each section. IBA1-positive microglia number per image and MBP levels were quantified in (B) and (C), respectively. MBP intensity was normalized to the average of AA samples. Data represent the means ± SEM. Statistical significance was analyzed by unpaired two-tailed Student’s *t* test (*n* = 6 to 7 for each genotype). *P < 0.05. (**D**) A model to illustrate the function of TMEM106B in microglia and myelination: Dysfunction of TMEM106B in microglia leads to decreased levels of TREM2, lysosomal abnormalities, and other defects; these alterations result in reduced microglia survival and proliferation in response to myelin debris, contributing to myelination deficits.

*TMEM106B* polymorphisms have been associated with brain aging and many neurodegenerative diseases (*55*). For the sentinel SNP rs1990622, allele rs1990622^A^ has been shown to act as a risk factor and rs1990622^G^ is protective in FTLD with *GRN* mutations and other neurodegenerative diseases. To determine whether the TMEM106B risk allele affects myelination and microglial activation in human samples, we analyzed MBP intensity and the number of IBA1-positive microglia in the corpus callosum region. Because of limited samples from control patients with different TMEM106B genotypes, we also analyzed sporadic FTLD-TDP cases without mutations in the main FTLD genes, *GRN* and *C9ORF72*. We found a significant myelin loss based on MBP staining intensities and reduced numbers of IBA1-positive microglia in samples with rs1990622^A/A^ genotypes compared to rs1990622^A/G^ genotypes (Fig. 10, A to C, and table S1). Unfortunately, we only have two samples with the rs1990622^G/G^ genotype, so we cannot perform statistical analysis for this group (table S1). Nevertheless, our data support the idea that TMEM106B affects microglial survival and myelination in humans, although our study is limited by the number of samples available.

## DISCUSSION

*TMEM106B* has been closely associated with brain aging and many brain disorders (*55*). However, the physiological and pathological functions of TMEM106B remain poorly understood. In this study, we revealed an unexpected function of TMEM106B in regulating microglial proliferation, survival, and activation in response to demyelination.

### TMEM106B regulates microglial dynamics in response to demyelination

Microglia play a crucial role in myelin growth and integrity (*64*) and become activated following demyelination and adopt different phenotypes (*50*, *65*–*67*). Activated microglia can cause cell damage by producing cytokines and nitric oxide. However, they are also involved in phagocytosis and the removal of myelin debris, which is essential for successful remyelination (*50*, *51*, *68*). Loss of microglia functions and a decrease in microglial numbers are often associated with leukodystrophies (*69*). In this study, we found several notable microglial phenotypes caused by TMEM106B deficiency, which include reduced microglial survival and increased microglial apoptosis (Figs. 2 and 6), as well as decreased microglial activation (Figs. 5 and 6) in response to demyelination and LPS stimulation. These microglial phenotypes are recapitulated in mice with TMEM106B specifically ablated in microglia (Fig. 7), supporting the notion that TMEM106B functions in microglia to support microglial survival in response to demyelination. In addition, microglia-specific deletion of TMEM106B leads to myelination defects (Fig. 7, F and G), consistent with the important role of microglia in the myelination process. Furthermore, we show that, in humans, TMEM106B risk alleles are associated with the decreased microglial number and myelin intensity in the corpus callosum region (Fig. 10, A and B).

Microglia self-renew through proliferation, especially during disease pathology, depending on the local need for microglia (*70*, *71*). Single-cell RNA-seq (scRNA-seq) analyses have revealed many functional subpopulations of microglia that coexist in the brain during the progress of different brain disorders, including homeostatic, DAM, and white matter–associated microglia (*72*, *73*). Although our current results show that TMEM106B is required for microglial survival in response to demyelination, TMEM106B deficiency does not have any obvious effect on microglial homeostasis in young mice under normal conditions (*28*). Upon CPZ removal, we found that *Tmem106b^−/−^* microglia can undergo cell proliferation to replenish the microglia population (fig. S6). In addition, microglial proliferation and activation have been observed in aged TMEM106B-deficient mice (*35*) and mice deficient in both TMEM106B and progranulin, another FTLD protein (*30*). Thus, TMEM106B is not essential for microglial activation and proliferation under these conditions, and it suggests a specific requirement of TMEM106B for microglial survival in response to demyelination.

Our results differ from a recent study examining microglial activation in TMEM106B-deficient mice in response to demyelination (*36*), in which increased microglial proliferation was found in TMEM106B-deficient mice. This might be caused by the differences in the TMEM106B-deficient mouse strains used. While TMEM106B protein is completely eliminated in our TMEM106B-deficient strain generated using CRISPR (*28*), residual expression of TMEM106B was found in the mouse strain used in their study (*74*).

### TMEM106B is important for proper lysosomal function in microglia

While the lysosomal pathway is slightly downregulated in *Tmem106b^−/−^* microglia under the normal condition (Fig. 3A), it is significantly increased in *Tmem106b^−/−^* microglia in response to CPZ-induced demyelination (Fig. 3B). Ablation of TMEM106B in the microglial cell line BV2 results in an increase in lysosomal pH and a decrease in the activities of lysosomal proteases CathD/E (Fig. 4, B and C). Increased levels of lysosomal proteins and enlarged lysosomes are frequently observed in *Tmem106b^−/−^* microglia in CPZ-treated mice (Fig. 4, D and E), supporting the notion that TMEM106B deficiency leads to severe lysosomal abnormalities in microglia upon myelin challenge. Lysosomal dysfunction, especially lysosomal membrane permeabilization, and the leakage of lysosomal hydrolase and substrates are associated with several cell death pathways and inflammatory responses (*75*, *76*), which might lead to the up-regulation of several immune signaling pathways and reduced survival of *Tmem106b^−/−^* microglia in CPZ-treated mice (Fig. 3B).

While the exact function of TMEM106B in the microglial lysosome remains to be determined, our data suggest that it is important for the proper maintenance of lysosomal pH (Fig. 4C). This is probably due to its association with lysosomal V-ATPase as previously reported (*26*, *28*). Also, it is interesting that recent bioinformatic studies have predicted TMEM106B as a lipid-binding protein (*77*). It is possible that TMEM106B is required for either sensing or metabolizing phagocytosed myelin lipids in microglia to maintain lysosomal homeostasis and microglial viability in response to the myelin challenge. Whether and how TMEM106B regulates lipid metabolism is still a total mystery. However, we do not detect any obvious changes in lipid droplet formation in *Tmem106b^−/−^* microglia in CPZ- or LPS-treated mice (fig. S7).

### TMEM106B regulates TREM2 levels

TREM2, a type I transmembrane receptor protein dominantly expressed in microglia, is involved in microglia proliferation, survival, migration, and phagocytosis (*41*, *43*, *44*). In this study, we found that TMEM106B loss results in decreased TREM2 levels. At the current stage, we can only speculate on how TMEM106B regulates TREM2 levels, and there are several possibilities. (i) TMEM106B might regulate TREM2 transcription since our RNA-seq analysis has shown decreased levels of TREM2 mRNA upon TMEM106B loss in microglia (fig. S5). (ii) TMEM106B could protect TREM2 from lysosome-dependent degradation. TREM2 has been shown to recycle from late endosomes/lysosomes to the Golgi compartment in a retromer-dependent manner (*78*). Since TMEM106B is mainly localized in the late endosome/lysosome compartment, and we have detected a weak interaction between TMEM106B and TREM2 when overexpressed (Fig. 9, E to G), it is possible that TMEM106B transiently interacts with TREM2 to promote its recycling through retromer in the late endosome/lysosome. Another possibility is that TMEM106B might protect TREM2 from the actions of lysosomal proteases, allowing more time for TREM2 recycling. (iii) In addition, TREM2 is cleaved extracellularly to generate soluble TREM2 fragments (*79*). TMEM106B loss could potentially lead to enhanced TREM2 cleavage, resulting in decreased TREM2 levels in the cell. It should be noted that TREM2 levels were reported to be slightly increased in TMEM106B-deficient mice in a previous study (*80*). While the discrepancy could be caused by differences in the *Tmem106b^-/-^* mouse strains used in the studies, it is also highly likely that TMEM106B is only required to maintain TREM2 levels under certain states of microglial activation, such as demyelination. Future work is needed to fully understand the role of TMEM106B in TREM2 regulation.

However, it is unlikely that decreased TREM2 levels alone are sufficient to cause microglial apoptosis in *Tmem106b^−/−^* mice since TREM2 levels are reduced to 32.5 to 78.3% of WT levels in *Tmem106b^−/−^* microglia under different conditions, and *Trem2^+/−^* mice do not show any obvious phenotypes in response to CPZ treatment (*81*). In addition, unlike *Trem2^−/−^* microglia, *Tmem106b^−/−^* microglia do not show defects in phagocytosis (Fig. 8, H to K). It is likely that lysosomal abnormalities combined with decreased TREM2 levels and other perturbations caused by TMEM106B loss as revealed by our RNA-seq analysis (Fig. 3) lead to decreased microglial survival and proliferation in *Tmem106b^−/−^* mice in response to CPZ treatment (Fig. 10D). The relative contribution of these pathways to microglial survival defects seen under TMEM106B-deficient conditions and the mechanisms by which TMEM106B regulates these pathways await to be dissected.

### TMEM106B functions in many different cell types to affect brain aging and health

Our study sheds light on the distinct function of TMEM106B in various brain cell types, contributing to the regulation of overall brain health. Previous work by us and others has shown that TMEM106B is essential for lysosome trafficking across the AIS segment in motor neurons (*30*, *31*) and aged Purkinje neurons (*33*, *35*) and TMEM106B is required for proper lysosome positioning and PLP trafficking in oligodendrocytes (*28*, *36*). In this study, we further show that TMEM106B is required for microglial survival in response to demyelination. Thus, loss of TMEM106B likely causes alterations in all these cell types, resulting in myelination deficits and neuronal dysfunction. Given that TMEM106B is intimately linked to brain aging, myelination disorder, and a vast number of neurodegenerative diseases (*55*), future research is warranted to fully elucidate TMEM106B functions in these different brain cell types. Additionally, investigating the impact of TMEM106B variants and the formation of TMEM106B C-terminal amyloid fibrils (*17*–*21*) on TMEM106B function will be critical for understanding how TMEM106B regulates brain aging and brain disorders.

## MATERIALS AND METHODS

### Antibodies and reagents

The following antibodies were used in this study: mouse anti-ACBD3 (Santa Cruz Biotechnology, sc-101277), mouse anti-APC (Millipore, OP80), goat anti-CathB (R&D Systems, AF965), goat anti-CathD (R&D Systems, AF1029), rat anti-CD68 (Bio-Rad, MCA1957), rabbit anti–cleaved caspase 3 (Cell Signaling Technology, 9661), mouse anti–galectin-3 (BioLegend, 126702), mouse anti–glyceraldehyde-3-phosphate dehydrogenase (Proteintech Group, 60004-1-Ig), mouse anti-GFAP (GA5) (Cell Signaling Technology, 3670S), rat anti-mouse LAMP1 (BD Biosciences, 553792), rabbit anti-IBA1 (Wako, 01919741), goat anti–AIF-1/Iba1 (Novus Biologicals, NB100-1028), rat anti-Ki67 (Invitrogen, 14-5698-82), mouse anti-PCNA (Cell Signaling Technology, 2586), mouse anti-MBP (Millipore, SMI-99), goat anti-OLIG2 (R&D Systems, AF2418), mouse anti-PLP (Millipore, MAB388), rat anti-TREM2 clone 5F4 (Millipore, MABN2321), rat anti-TREM2 (R&D Systems, MAB17293), goat anti-hTREM2 (R&D Systems, AF1828), and sheep anti-TREM2 (R&D Systems, AF1729). Rabbit anti-TMEM106B antibodies have been previously characterized (*22*).

The following reagents were also used in the study: Dulbecco’s modified Eagle’s medium (DMEM) (Cellgro, 10-017-CV), Hanks’ balanced salt solution (HBSS) (Cellgro, 21-020-CV), DMEM/Ham’s F-12 (Cellgro, 10-092-CV), 0.25% trypsin (Corning, 25-053-CI), Autofluorescence Quencher (Biotium, 23007), Odyssey blocking buffer (LI-COR Biosciences, 927-40000), a protease inhibitor (Roche, 05056489001), Pierce Bicinchoninic Acid (BCA) Protein Assay Kit (Thermo Fisher Scientific, 23225), and optimal cutting temperature (O.C.T.) compound (Electron Microscopy Sciences, 62550-01).

### Mouse strains and treatment

C57BL/6 mice were obtained from the Jackson Laboratory. TMEM106B knockout mice (Δ341bp) were generated and crossed back to the C57BL/6 background for more than 10 generations as previously described (*28*). To generate conditional *Tmem106b^flox/flox^* mice*, Tmem106b^tm2a(KOMP)Wtsi^* mice from the Knockout Mouse Project (KOMP) (C57BL/6N-*Tmem106b*^tm2a(KOMP)Wtsi^/MbpMmucd, RRID:MMRRC_050092-UCD), which contains FLP recombination target (FRT) sites flanking a lacZ gene trap cassette inserted between exons 3 and 4 and loxP sites flanking exon 4 of the *Tmem106b* gene, were mated with β-actin-Flpe mice (www.jax.org/strain/003800) (*82*) on the C57BL/6 background (B6n8, a gift of J. Sun and R. Ray, Baylor College of Medicine) to delete the lacZ gene trap (fig. S4). *Tmem106b^flox/flox^* mice were then crossed with mice with a tamoxifen-inducible Cre-mediated recombination system (Cre-ERT2) driven by a *Cx3cr1* promoter (www.jax.org/strain/021160) (*63*). *Cx3cr1^+/CreER^ Tmem106b ^flox/flox^* mice were fed with tamoxifen at 4 weeks of age to delete TMEM106B in microglia. Tamoxifen (Sigma-Aldrich, T5648, 10 mg/ml) was dissolved in filter-sterilized corn oil by incubating overnight at 37°C. The solution was protected from light and administered to mice (75 mg/kg) via oral gavage every other day seven times. *Cx3cr1^+/CreER^* mice were used as controls. Mixed male and female mice were used for this study. All animals (one to six adult mice per cage) were housed in a 12-hour light/12-hour dark cycle in the Weill Hall animal facility at Cornell University. All animal procedures have been approved by the Institutional Animal Care and Use Committee at Cornell University.

CNS demyelination was induced by supplementing the diet of 10-week-old mice with 0.2% (w/w) CPZ [bis (cyclohexanone) oxaldihydrazone, Sigma-Aldrich] in powdered rodent chow (*83*). The rodent chow (5 g per mouse per day) was replaced every other day for 3 or 5 weeks for the remyelination period, and the mice were fed with the CPZ-containing diet for 5 weeks and then returned to normal chow for 3 weeks. Untreated control mice were fed normal crushed chow for 5 weeks.

For intracerebroventricular injection, mice were anesthetized with isoflurane following institutional guidelines and then placed into a stereotaxic instrument (Stoelting Co.). A small hole (2-μm diameter) in the skull was drilled using a dental drill. A 26-gauge stainless steel guide cannula (Plastics One Inc.) was implanted in the right and left lateral ventricle (anteroposterior, +0.3 mm; lateral, ±1.0 mm; dorsoventral, 2.5 mm). A total of 5 μg of LPS dissolved in 2 μl of saline was injected using a stepper-motorized microsyringe (Stoelting) at a rate of 0.2 μl/min. The needle was left in place for at least 5 min after injection. After the operation, the skin was then sutured, and mice were treated with buprenorphine (0.1 mg/kg) every 8 hours for 24 hours to control pain. Mice were perfused, and brain tissues were collected 72 hours after the LPS injection.

### DNA constructs

TREM2 complementary DNA (cDNA) in the pDONR221 vector was obtained from DNASU Plasmid Repository and then cloned into the pcDNA3.1mychis A (+) vector (Invitrogen) using Bam HI and Xho I to generate TREM2-mychis fusion expression construct. pDONR221-TREM2 was recombined with pLenti-CMV-puro destination vector (Addgene) in a gateway reaction to generate the pLenti-CMV-TREM2 construct. CD68 cDNA in the pDONR-Dual vector was obtained from DNASU and cloned in the pEFP-N2 vector using Hind III and Eco RI to generate the CD68-GFP construct. Human TMEM106B cDNA in the pEGFP vector, pCMV3XFLAG7.1, and pCMV-Sport6 was obtained as previously described (*22*).

### Cell culture

BV2, HEK293T, and HeLa cells were maintained in DMEM (Cellgro) supplemented with 10% fetal bovine serum (FBS; Gibco) and 1% penicillin-streptomycin (Invitrogen) in a humidified incubator at 37°C and 5% CO_2_. Cells were transiently transfected with polyethyleneimine as described previously (*84*).

TREM2-expressing lentiviruses were generated by transfecting lentiviral vectors together with pMD2.G and psPAX2 plasmids into HEK293T cells. Viral supernatant was collected 3 days after transfection and used to infect HeLa cells. Cells were then selected with puromycin (2 μg/ml) to obtain HeLa cells stably expressing TREM2.

BV2 cells with TMEM106B deletion or controls were generated by infecting the cells with lentivirus expressing Cas9 and guide RNAs (TACTTCACCTCAATAGATCG) targeted to mouse Tmem106b exon 3. Cells were selected with puromycin (2.5 μg/ml) 2 days after infection, and the knockout was confirmed by Western blot and immunostaining.

Primary microglia were prepared from postnatal days 0 to 2 of WT and *Tmem106b^−/−^* pups. Briefly, cortices were rapidly dissected from the brain in 2 ml of HBSS at 4°C followed by digestion with 0.25% trypsin. The cells were maintained in DMEM/F12 with 10% FBS at 37°C in a 5% CO_2_-humidified atmosphere. Granulocyte-macrophage colony-stimulating factor (GM-CSF) was added at 5 ng/ml to stimulate microglial proliferation. After 14 days in culture, the flasks were shaken to separate microglia from mixed glial cultures. The purity of primary microglia was greater than 95% validated by IBA1 immunostaining. After 24 hours, the cultured medium was replaced with a fresh medium for 1 to 2 days until used for experiments.

### Cell viability assay and TUNEL staining

Primary microglial cells were seeded in 96-well plates at a density of 3 × 10^4^ cells per well for 24 hours and treated with GM-CSF (5 ng/ml), myelin (10 μg/ml), or LPS (100 ng/ml) for an additional 24 hours. Cells were fixed and stained with Hoescht. The number of cells in each well was measured by ImageXpress (Molecular Devices).

The numbers of apoptotic cells were determined by TUNEL. Briefly, cells were washed with phosphate-buffered saline (PBS), fixed in 4% paraformaldehyde (PFA) for 15 min, washed three times again with PBS, and then stained with TUNEL according to the manufacturer’s instructions (CF Dye TUNEL Assay Apoptosis Detection Kits, Biotium). Hoechst was used to stain cell nuclei. The images were captured under a Leica DMi8 inverted microscope, and the positive apoptosis cells were quantified using ImageJ.

### Phagocytosis assay

Primary microglial cells were incubated with the fluorescence-labeled Latex beads (Polysciences.com) for 2 hours. After washing with PBS, cells were fixed in 4% PFA and stained with lysosomal marker LAMP1 and Hoechst. The fluorescent signals were captured under a Leica DMi8 inverted microscope and analyzed using ImageJ.

Myelin was sonicated before administration to the cells. For the myelin treatment, the cells were washed twice and incubated with purified myelin (10 μg of myelin per cm^2^) in serum-free media to remove the effect of the serum lipoproteins. Two hours later, the cells were washed and left for the experiment time. The number of intracellular myelin particles was determined by MBP staining. The intensity of MBP-positive particles was measured and divided by the cell number as determined by Hoechst staining. Cells (100 to 300) were analyzed per experiment.

### Lysosome pH measurement

Bv2 cells were seeded at a density of 10,000 cells per well in a 96-well glass bottom plate (Cellvis, P96-1.5H-N). Fluorescein-tetramethylrhodamine dextran (70 kDa; Invitrogen D1951) with a stock concentration of 25 mg/ml was diluted to a working concentration of 1 mg/ml in growth media. After overnight incubation, cells were washed and chased in regular growth media for 2 hours. Live cell imaging was carried out with cells in the medium buffered with 20 mM Hepes (pH 7.4) under a CSU-X spinning disc confocal microscope (Intelligent Imaging Innovations) prewarmed to 37°C with an HQ2 charge-coupled device (CCD) camera (Photometrics) using 100× objectives. Cells were then fixed using 4% PFA, equilibrated in pH calibration standard solutions (50 mM PBS, 50 mM tris maleate, and 40 mM methylamine, adjusted to pH 4, 4.5, 5, and 5.5 using HCl or NaOH) for 10 min, and imaged using the same microscope. The green [fluorescein isothiocyanate (FITC)] and red [tetramethyl rhodamine isothiocyanate (TRITC)] fluorescent intensities per cell were measured using the ImageJ software, and the ratio was calculated for both the experimental group and the calibration group. FITC/TRITC ratios were plotted against pH values and fitted to an exponential decay function. Corresponding pH values for each ratio were interpolated from the calibration curve. The experiments were independently repeated three times.

### Brain tissue

Human brain tissues were obtained from the Neurodegenerative Disease Brain Bank at the University of California, San Francisco. Authorization for autopsy was provided by the patients’ next of kin, and procedures were approved by the UCSF Committee on Human Research. Neuropathological diagnoses were made following consensus diagnostic criteria (*85*, *86*). Cases were selected on the basis of neuropathological diagnosis and genetic analysis. Brain tissues were obtained from individuals with minimal age-related neurodegenerative changes or individuals diagnosed with FTLD-TDP type A without *GRN* or *C9orf72* mutations. Detailed information is provided in table S1.

### Immunofluorescence staining, image acquisition, and analysis

For paraffin-embedded human brain samples, 8-μm-thick sections including the corpus callosum were deparaffinized with xylene and ethanol. Antigen retrieval was performed by microwaving in citrate buffer (pH 6.0) for 20 min. For mouse brain immunostaining, mice were transcardially perfused with PBS and 4% PFA. The brains were removed and continuously postfixed overnight in 4% PFA at 4°C. After being dehydrated, coronal brain sections including the cortex, corpus callosum (overlying the fornix approximately between bregma 0.86 and 0.14 mm), or hippocampus (approximately between bregma −1.34 and −2.3 mm) were cut using a cryostat (Leica, Heidelberg, Germany) and processed for immunostaining. Brain sections were blocked and permeabilized with 0.1% saponin in Odyssey blocking buffer before incubating with primary antibodies overnight at 4°C. The next day, sections were washed with PBS three times followed by incubation with secondary fluorescent antibodies and Hoechst at room temperature for 2 hours. The slides were then mounted using a mounting medium (Vector Laboratories). Lower-magnification images were captured by 20× objectives on a Leica DMi8 inverted microscope. High-resolution images were acquired on a CSU-X spinning disc confocal microscope (Intelligent Imaging Innovations) with an HQ2 CCD camera (Photometrics) using 63× or 100× objectives. Five to 10 random fields of the corpus callosum region were examined for human brain sections. Ten to 15 different random images were captured from the cortex, corpus callosum, or hippocampus region of mouse brain sections.

For the quantitative analysis of MBP, PLP, IBA1, and GFAP levels in the brain sections, the fluorescence intensity was measured directly using ImageJ software (National Institutes of Health, Bethesda, MD, USA) after a threshold application. The protein levels were determined by the total fluorescence signals. For the quantitative analysis of Galectin-3, TREM2, CD68, LAMP1, and CathD levels in microglia, the IBA1^+^ microglia were selected using the region of interest (ROI) tool after the data channels were separated (Image\Color\Split Channels). Next, galectin-3, TREM2, LAMP1, CathD, or CD68 signals within the IBA1 ROI were selected (Analyze\tools\ROI manager) and measured. The number of IBA1^+^ microglia, Ki67^+^IBA1^+^ microglia, PCNA^+^IBA1^+^ microglia, cleaved caspase 3^+^ (C-casp3^+^) IBA1^+^ microglia, and APC^+^OLIG2^+^ cells was counted using the “analyze particles” function of ImageJ after a threshold application. Proliferating microglia are defined by colocalization of Ki67 or PCNA with the Hoechst nuclei signals in IBA1^+^ cells. Three to five nonadjacent brain sections per mouse were used for quantification. A total of 40 to 60 microglia per group were analyzed from three to four independent mice per group. The mean from all sections was used to be representative of each mouse. To determine the % Ki67^+^IBA1^+^/IBA1^+^ cells, % PCNA^+^IBA1^+^/IBA1^+^ cells, % C-casp3^+^IBA1^+^/IBA1^+^ cells, and % APC^+^OLIG2^+^/OLIG2^+^ cells, the number of double-labeled cells was divided by the total number of IBA1^+^ microglia or OLIG2^+^ cells and then multiplied by 100%.

Microglia morphometric analysis was performed using RI [4π × cell area/(cell perimeter)^2^] as previously described (*58*, *59*). Cell area and perimeter were calculated with ImageJ. Ten microglia were analyzed for mice fed with a normal diet. A total of 40 to 70 cells per group were analyzed from three to five independent mice per group for CPZ-treated mice at each time point.

Cultured cells were grown on coverslips in 24-well plates. Cells were fixed in 4% PFA, permeabilized, and blocked with 0.05% saponin in Odyssey blocking buffer. Primary antibodies were incubated at 4°C overnight, which was detected by secondary fluorescent antibodies. Hoechst was used to stain cell nuclei. Coverslips were mounted using a mounting medium (Vector laboratories). Fluorescence was visualized under a confocal microscope.

### Western blot analysis

Mice were perfused with 1× PBS, and tissues were dissected and snap-frozen with liquid nitrogen and kept at −80°C. On the day of the experiment, frozen tissues were thawed and homogenized on ice with a bead homogenizer (Moni International) in ice-cold radioimmunoprecipitation assay (RIPA) buffer [150 mM NaCl, 50 mM Tris-HCl (pH 8.0), 1% Triton X-100, 0.5% sodium deoxycholate, and 0.1% SDS] with proteinase and phosphatase inhibitors. Cultured cells were lysed in ice-cold RIPA buffer with proteinase and phosphatase inhibitors. Supernatants were collected after centrifugation at 14,000*g* for 15 min at 4°C. Protein concentrations were determined via BCA assay and then standardized. Equal amounts of protein from each sample were run on 12 or 15% polyacrylamide gels and then transferred to the polyvinylidene difluoride membrane (Millipore, Billerica, MA, USA). Membranes were blocked with either 5% nonfat milk in PBS or Odyssey Blocking Buffer (LI-COR Biosciences) for 1 hour and then incubated with primary antibodies, rocking overnight at 4°C. Membranes were then washed with tris-buffered saline with 0.2% Tween-20 three times for 5 min each and incubated with fluorescently tagged secondary antibodies (LI-COR Biosciences) for 1 hour at room temperature. Membranes were scanned after three washes using the Odyssey Infrared Imaging System (LI-COR Biosciences). Densitometry was performed with Image Studio (LI-COR Biosciences).

### Coimmunoprecipitation

Cells were lysed in a cold, near-neutral pH solution containing 150 mM NaCl, 50 mM tris-HCl (pH 7.5), 1% Triton X-100, 0.1% deoxycholic acid, and 1× protease inhibitors (Roche). After centrifugation at 14,000*g*, for 15 min, at 4°C, supernatants were transferred to clean tubes on ice, to which GFP-Trap beads (ChromoTek) were added and then rocked for 3 hours at 4°C. Immunocomplexes and 10% amounts of cell lysates for immunoprecipitation (as input) were analyzed by Western blot with antibodies against Myc or GFP to recognize immunoprecipitated proteins.

### CathD/E activity assay

BV2 cells were lysed in lysis buffer [20 mM sodium acetate (pH 5.3), 150 mM NaCl, and 1% Triton X-100]. Ten micrograms of lysates was incubated at 37°C for 15 min in 100 μl of assay buffer [50 mM sodium acetate (pH 5.5), 0.1 M NaCl, 1 mM EDTA, and 0.2% (v/v) Triton X-100] in the presence of the fluorogenic substrate [MOCAc-GKPILF~FRLK(Dnp)-D-R-NH2] (Calbiochem, catalog no. 219360). The fluorescence released as a result of CathD/E proteolytic activity was read at 340 nm (excitation) and 420 nm (emission).

### RNA-seq analysis

CD11b^+^ microglia were isolated from adult mouse brains using magnetic-activated cell sorting according to published protocols (*87*, *88*). The purity of microglia was verified by immunoblotting with microglia marker IBA1, astrocyte marker GFAP, neuronal marker NeuN, and oligodendrocyte marker Olig2 (fig. S1). Total RNA was extracted from isolated microglia using Trizol (Thermo Fisher Scientific). RNA quality was checked using nanodrop, gel electrophoresis, and Agilent Fragment Analyzer. RNA-seq libraries were generated by the Cornell TREx Facility using the NEBNext Ultra II Directional RNA Library Prep Kit (New England Biolabs) using 700-ng input total RNA per sample. At least 20 million reads (2 × 150 nt PE) were generated on a NovaSeq (Illumina). Reads were trimmed to remove low-quality and adaptor sequences with TrimGalore (a wrapper for cutadapt and fastQC), requiring a minimum trimmed length of 50 nucleotides (nt). Reads that passed quality control were aligned to reference genome (mouse GRCm38/mm10) (*89*) using STAR (*90*), using “--quantMode GeneCounts” to output counts per gene. SARTools (*91*) and DESeq2 (*92*) were used to generate normalized counts and statistical analysis of differential gene expression. Genes with normalized counts ≥50 were included in the following analysis. Genes with FDR control *P* value ≤0.05 and log fold change ≥0.5 were identified as DEGs. Enrichment analysis using Kyoto Encyclopedia of Genes and Genomes (KEGG) pathway analyses (KEGG) gene sets was performed using GSEA (*93*). GSEA was applied using the raw counts of all genes expressed with recommended default settings (1000 permutations and a classic scoring scheme). The FDR was estimated to control the false-positive finding of a given normalized enrichment score by comparing the tails of the observed and null distributions derived from 1000 gene set permutations. The gene sets with an FDR < 0.25 or nominal *P* value <0.05 were considered as significantly enriched. Heatmap of gene expression changes was generated by using Heatmapper (*94*).

### Statistical analysis

All statistical analyses were performed using GraphPad Prism 8. All data are presented as means ± SEM. Statistical significance was assessed by unpaired Student’s *t* test or one-sample *t* test (for two-group comparison) or one-way analysis of variance (ANOVA) or two-way ANOVA tests with Bonferroni’s multiple comparisons (for multiple comparisons). *P* values less than or equal to 0.05 were considered statistically significant. *P < 0.05, **P < 0.01, ***P < 0.001, and ****P < 0.0001.

## Supplementary Materials

### This PDF file includes:

Figs. S1 to S7 Table S1 Legends for datasets S1 to S3

### Other Supplementary Material for this manuscript includes the following:

Datasets S1 to S3

## Notes

View/request a protocol for this paper from *Bio-protocol*.
