# Supplementary material for: TMEM106B regulates microglial proliferation and survival in response to demyelination
Source: Sci Adv. 2023 May 5;9(18):eadd2676. doi: 10.1126/sciadv.add2676 (PMC10162677; doi:10.1126/sciadv.add2676)
Supplement: Supplementary file 1 — Figs. S1 to S7 Table S1 Legends for datasets S1 to S3 [file sciadv.add2676_sm.pdf]

Science Advances
AAAS

Supplementary Materials for

TMEM106B regulates microglial proliferation and survival in response to demyelination

Tingting Zhang *et al.*

Corresponding author: Fenghua Hu, fh87@cornell.edu

Sci. Adv. **9**, eadd2676 (2023)
DOI: 10.1126/sciadv.add2676

The PDF file includes:

Figs. S1 to S7
Table S1
Legends for datasets S1 to S3

Other Supplementary Material for this manuscript includes the following:

Datasets S1 to S3

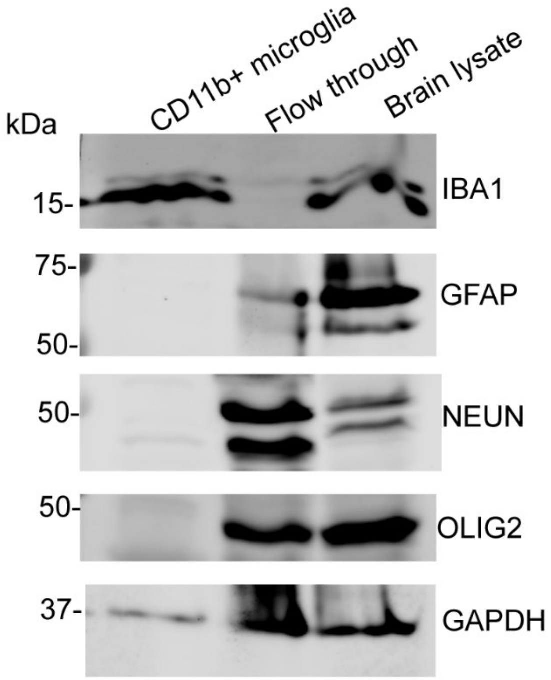

Figure S1: Western blot to demonstrate the purity of isolated microglia from adult brain. Lysates from CD11b beads isolated microglia, the flow-through fraction or the whole brain were blotted with antibodies indicated.

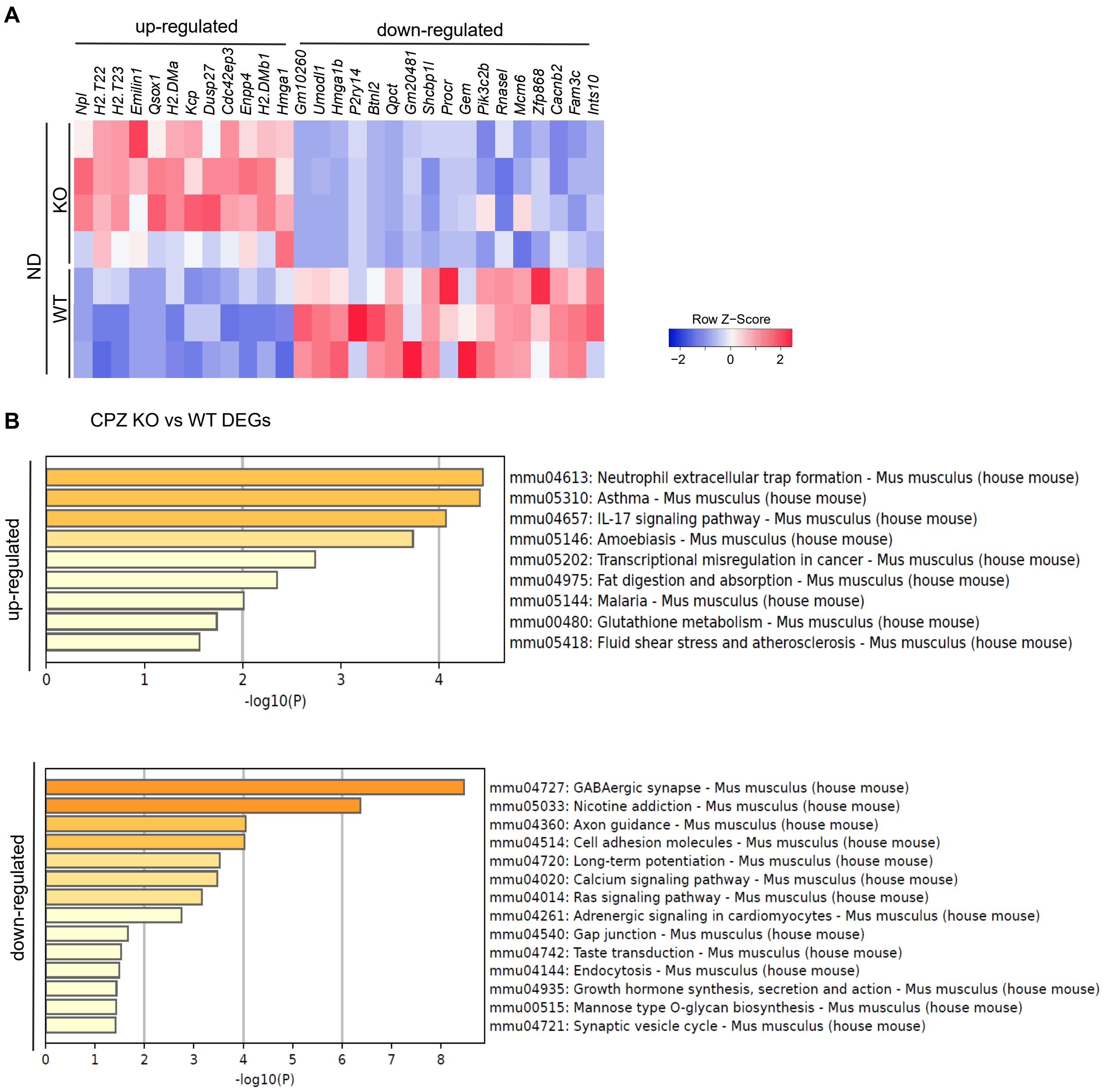

Figure S2: Analysis of DEGs in untreated and treated conditions. (A) Heatmap analysis of DEGs DEGs with normalized counts >50 and FDR<0.05 (Dataset S2) between untreated WT and KO microglial samples. **(B)** Pathway analysis of enriched pathways using DEGs with normalized counts >50 and FDR<0.05 (Dataset S2) between CPZ treated WT and KO microglial samples. 164 DEGs upregulated in the KO samples and 703 DEGs downregulated in the KO samples were analyzed using the KEGG database in Metascape (https://metascape.org) with p<0.05 as the cutoff.

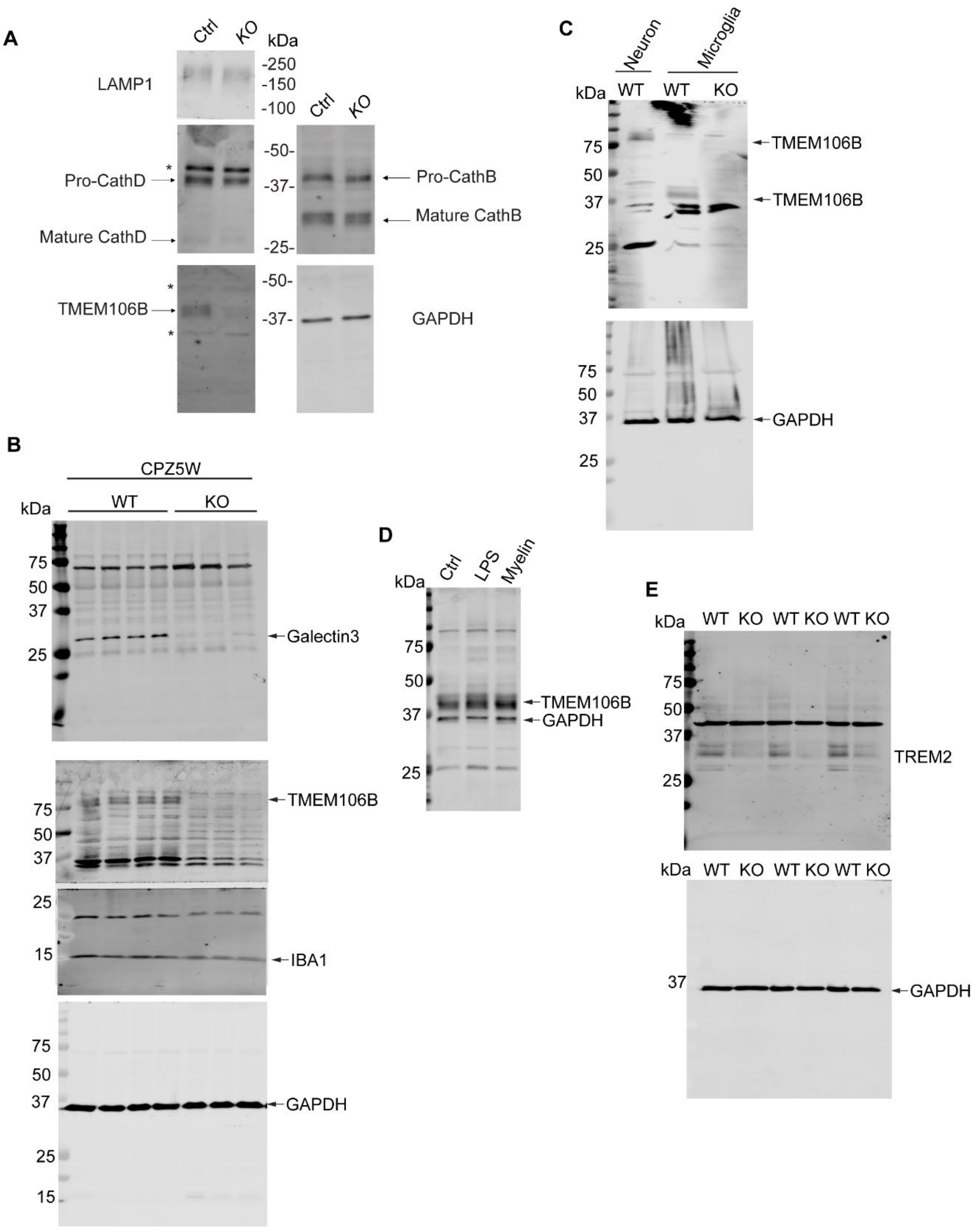

Figure S3: Full gel western blot for data shown in Fig. 4A (A), Fig. 5E (B), Fig. 8A (C), Fig. 8C (D), and Fig. 9C (E).

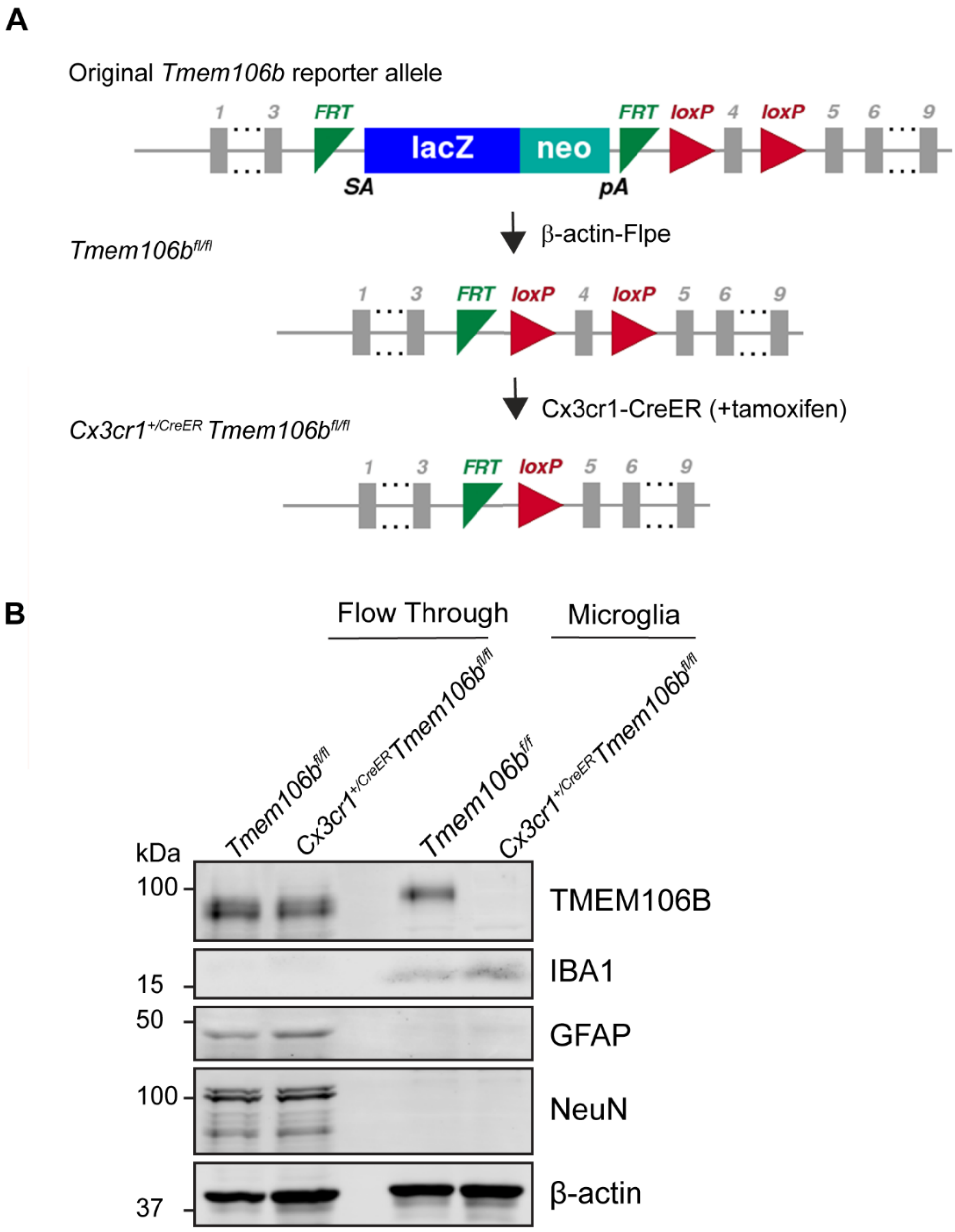

Figure S4: Generation of microglia specific ablation of TMEM106B. (A) Schematic drawing to illustrate the generation of microglia-specific ablation of TMEM106B. The diagram is adapted from the images on the KOMP website. **(B)** CD11b+ microglia were isolated from *Tmem106b* $^{flox/flox}$ and *Cx3cr1*$^{+/CreER}$ *Tmem106b* $^{flox/flox}$ mice. Lysates from isolated microglia and the flow-through fraction were blotted with antibodies indicated.

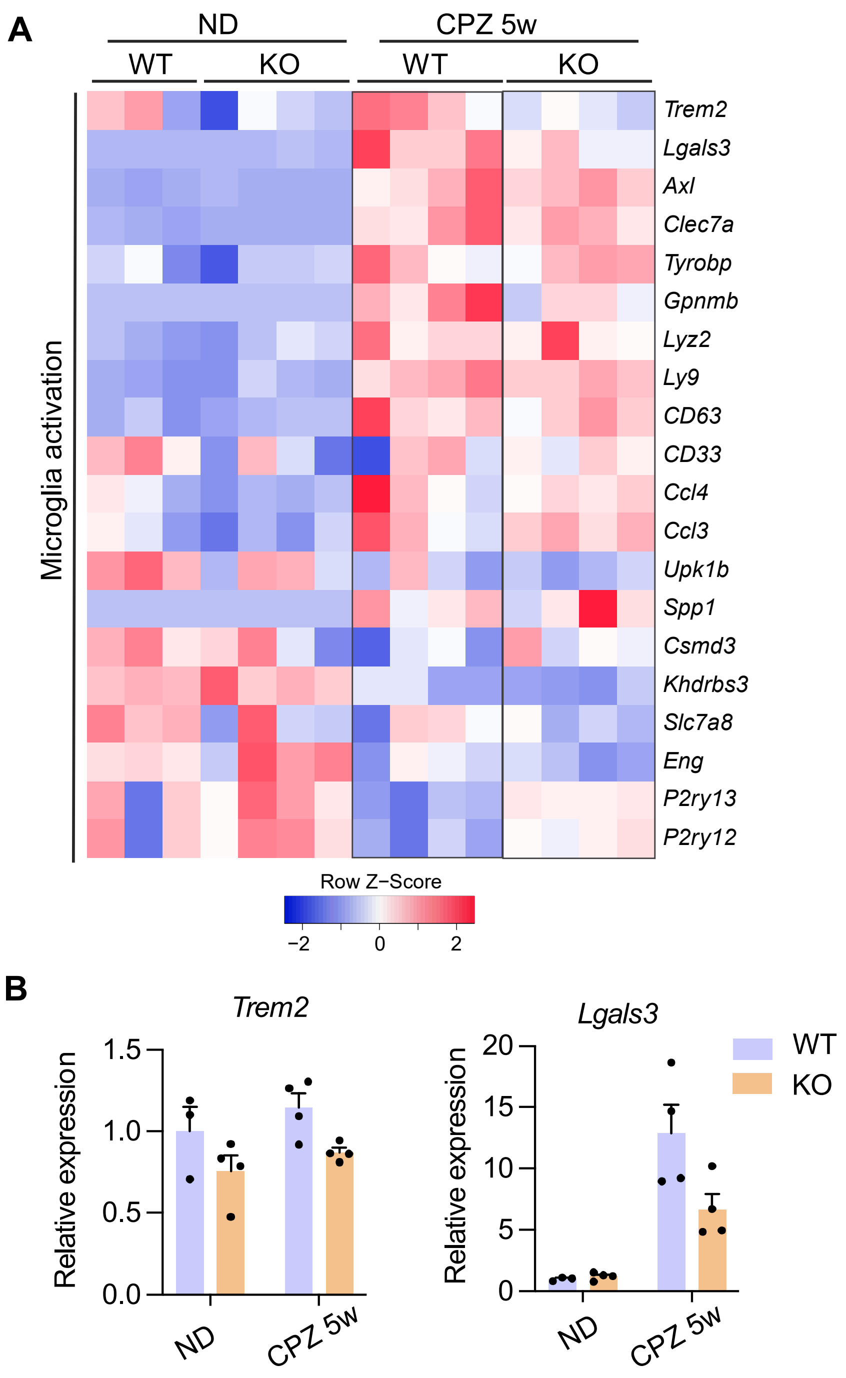

Figure S5: TMEM106B deficiency results in reduced Trem2 and Galectin-3 expression in microglia. (A) Heatmap showing gene expression changes of DAM microglial markers between CPZ treated WT and KO microglial samples. The list of DAM genes is based on published data (*73, 82*). **(B)** Normalized counts from the RNA seq data were normalized to untreated WT control and plotted.

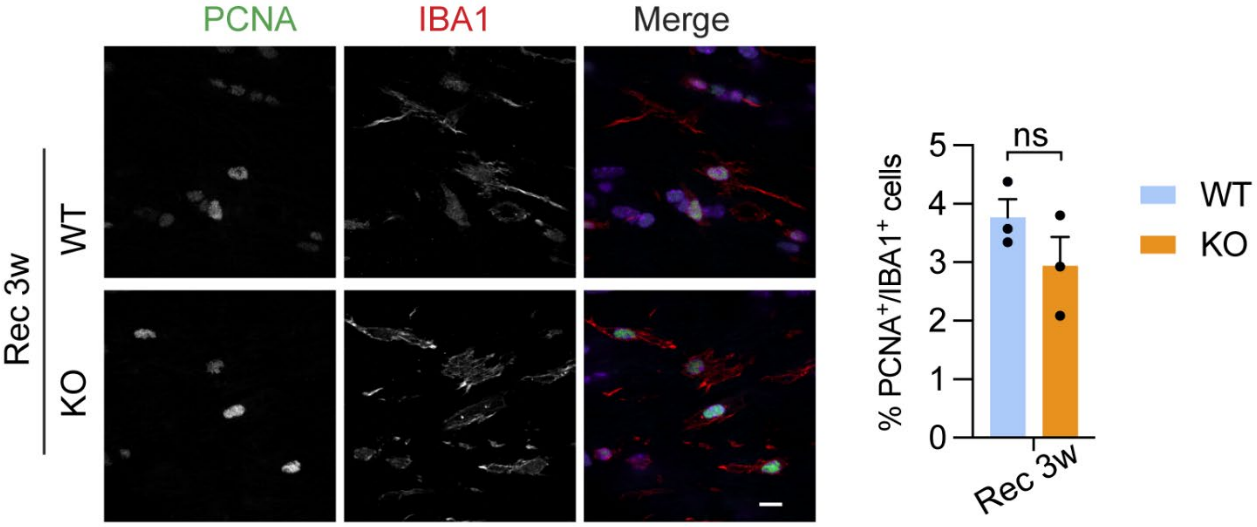

Figure S6: TMEM106B deficiency does not affect microglial proliferation after cuprizone withdrawal. WT and KO mice were fed with cuprizone-containing chow for 5 weeks and a normal diet for additional 3 weeks after cuprizone removal (Rec 3w). Brain sections were stained with PCNA and IBA1 antibodies. Representative images from the corpus callosum region were shown. Scale bar = 10 µm. The percentage of PCNA+ microglia was quantified. Data represent the mean ± SEM. Statistical significance was analyzed by unpaired two-tailed Student's t-test (n = 3 mice per group). ns, not significant.

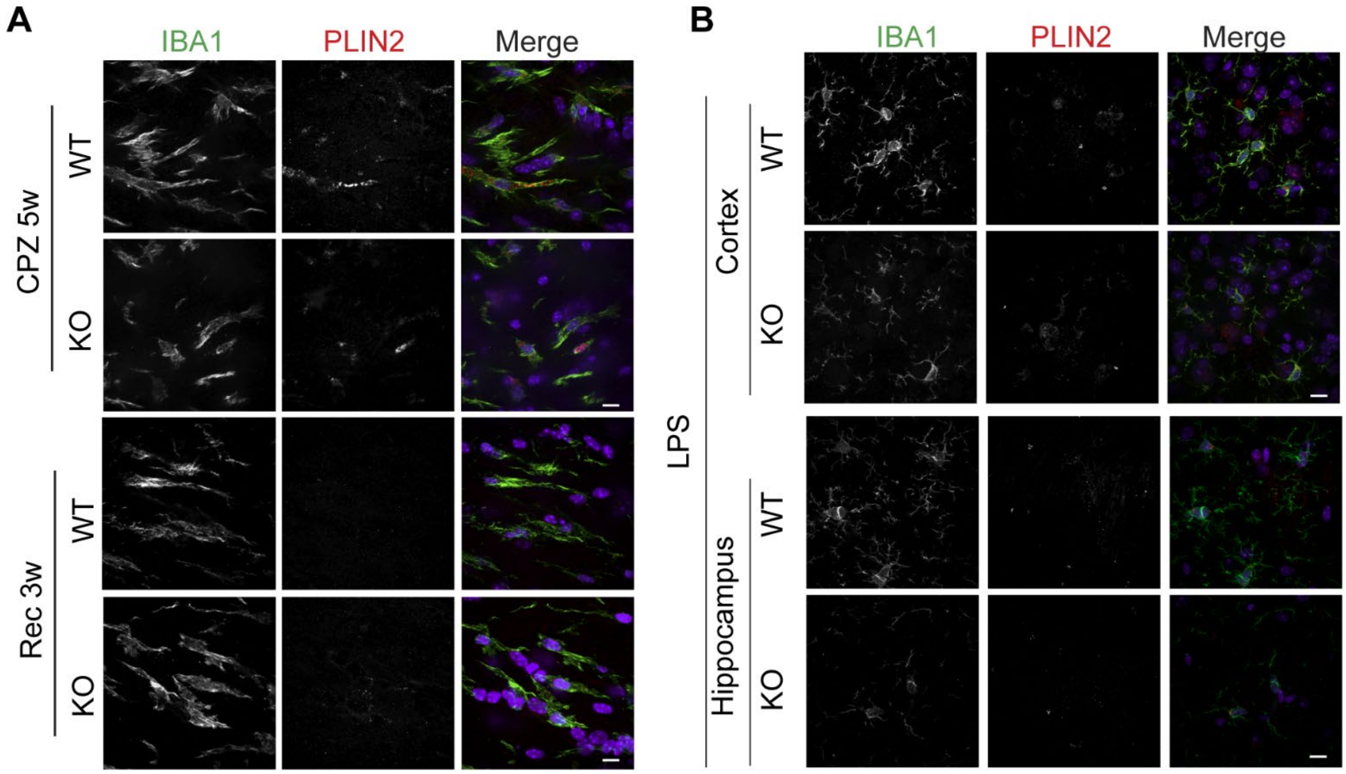

Figure S7: TMEM106B deficiency does not affect lipid droplet formation in microglia. Brain sections from CPZ or LPS treated mice were stained with PLIN2 and IBA1 antibodies. Representative images from the corpus callosum region (A) or cortex and hippocampus region (B) were shown. Scale bar = 10 μm.

Table S1. List of post-mortem samples used in the study.

Case #	Age at death	Sex	Clinical	Primary Neuropath Dx	TMEM106B genotype	Microglia number (per image)	MBP intensity
Control 1	78	F	control	None	AG	164.27	1.00
Control 2	76	M	control	None	AG	130.57	0.57
Control 3	91	M	control	None	AG	123.25	0.77
Control 4	92	F	control	None	AG	127.75	1.05
Control 5	84	F	control	None	AG	138.33	0.57
Control 6	91	M	control	None	AA	108	0.39
Control 7	86	F	control	None	GG	142.75	1.02
Control 8	86	F	control	None	GG	159.33	0.51
FTLD 1	72	M	PPA-mixed	FTLD-TDP-A	AA	129.5	0.37
FTLD 2	70	M	bvFTD	FTLD-TDP-A	AA	136	0.47
FTLD 3	83	M	PPA, unspecific	FTLD-TDP-A	AG	260.33	0.48
FTLD 4	78	F	nfvPPA	FTLD-TDP-A	AA	83.83	0.26
FTLD 5	66	F	CBS	FTLD-TDP-A	AA	91.4	0.73
FTLD 6	73	F	bvFTD	FTLD-TDP-A	AA	116.83	0.47
FTLD 7	78	F	AD vs. FTD	FTLD-TDP-A	AG	191.4	0.75

AD, Alzheimer's disease; bvFTD, behavioral variant frontotemporal dementia; CBS, Corticobasal syndrome; FTLD, Frontotemporal Lobar Degeneration; nfvPPA, nonfluent variant primary progressive aphasia; PPA, primary progressive aphasia. MBP staining intensity was normalized to Control 1 (set as 1.00).

Dataset S1: List of normalized counts identified in the RNA seq analysis for purified WT and KO adult microglia from ND and CPZ 5W conditions.

Dataset S2: List of DEGs with normalized counts >50 and FDR<0.05.

Dataset S3: List of genes in the selected KEGG pathways identified using GSEA analysis comparing CPZ treated WT and KO microglial samples.
